# Rapid Evolution of Phenotypic Plasticity and Shifting Thresholds of Genetic Assimilation in the Nematode *Caenorhabditis remanei*

**DOI:** 10.1534/g3.114.010553

**Published:** 2014-04-11

**Authors:** Kristin L. Sikkink, Rose M. Reynolds, Catherine M. Ituarte, William A. Cresko, Patrick C. Phillips

**Affiliations:** * Institute of Ecology and Evolution, University of Oregon, Eugene, Oregon 97403-5289; † Department of Biology, William Jewell College, Liberty, Missouri 64068

**Keywords:** genetic assimilation, experimental evolution, natural selection, heat shock proteins, heat stress, hormesis

## Abstract

Many organisms can acclimate to new environments through phenotypic plasticity, a complex trait that can be heritable, subject to selection, and evolve. However, the rate and genetic basis of plasticity evolution remain largely unknown. We experimentally evolved outbred populations of the nematode *Caenorhabditis remanei* under an acute heat shock during early larval development. When raised in a nonstressful environment, ancestral populations were highly sensitive to a 36.8° heat shock and exhibited high mortality. However, initial exposure to a nonlethal high temperature environment resulted in significantly reduced mortality during heat shock (hormesis). Lines selected for heat shock resistance rapidly evolved the capacity to withstand heat shock in the native environment without any initial exposure to high temperatures, and early exposure to high temperatures did not lead to further increases in heat resistance. This loss of plasticity would appear to have resulted from the genetic assimilation of the heat induction response in the noninducing environment. However, analyses of transcriptional variation via RNA-sequencing from the selected populations revealed no global changes in gene regulation correlated with the observed changes in heat stress resistance. Instead, assays of the phenotypic response across a broader range of temperatures revealed that the induced plasticity was not fixed across environments, but rather the threshold for the response was shifted to higher temperatures over evolutionary time. These results demonstrate that apparent genetic assimilation can result from shifting thresholds of induction across environments and that analysis of the broader environmental context is critically important for understanding the evolution of phenotypic plasticity.

Organisms regularly experience changes in their environments to which they must adapt to survive. As a consequence, many organisms have evolved the capacity to respond to stressful changes in environmental conditions by coherently altering their phenotypic attributes. This phenotypic plasticity, defined as the ability of a genotype to consistently produce an alternate phenotype in response to environmental variation (Bradshaw 1965), is known to be an important contributor to fitness in many organisms, including bacteria (Justice *et al.* 2006; Kümmerli *et al.* 2009; Butler *et al.* 2010), plants (Dudley and Schmitt 1996; Huber 1996; Agrawal 1998; Harder and Johnson 2005), and animals (Parejko and Dodson 1991; Warkentin 1995; Aubret *et al.* 2004; Charmantier *et al.* 2008; Cheviron *et al.* 2013).

Like any other character of organisms, phenotypic plasticity itself has a genetic basis that can change in response to evolutionary processes. One extreme evolutionary outcome of adaptation to a novel environment is the complete loss of ancestral phenotypic plasticity after selection, which is known as genetic assimilation (Waddington 1953, 1956). More generally, adaptation in one environment can lead to changes in phenotypic plasticity across other environments due to genetic correlations generated by the pleiotropic effects of genes responding to both environments or by genetic linkage of genes with independent effects within each environment. Quantitative genetic models (Via and Lande 1985; Gomulkiewicz and Kirkpatrick 1992; Gavrilets and Scheiner 1993) predict that these genetic correlations across environments determine how plasticity across environments evolves over time.

Although there has been renewed interest in the evolution of phenotypic plasticity and its importance for affecting the rate and direction of evolution of populations experiencing novel environments (Matesanz *et al.* 2010; Pfennig *et al.* 2010; Moczek *et al.* 2011), it is still unclear how fast phenotypic plasticity can evolve or what the molecular genetic basis underlying this evolution actually is. Except for a few classes of genes, most notably the heat shock proteins (hsps), which have been well-characterized for their role in regulating physiological responses to stress (Lindquist and Craig 1988) and acting as a capacitor for environmentally sensitive variation (Rutherford and Lindquist 1998; Cowen and Lindquist 2005; Sangster *et al.* 2008; Jarosz and Lindquist 2010; Rohner *et al.* 2013), little is known about where genetic variation for phenotypic plasticity resides in organisms’ genomes. For example, from a mechanistic standpoint, it is not known to what extent the evolution of phenotypic plasticity occurs primarily via changes in frequencies of alleles affecting protein-coding regions of genes as compared to regulatory changes affecting differential expression of genes. Dissecting the genetic basis of evolutionary change in phenotypic plasticity is particularly important because both the rate of evolution of phenotypic plasticity itself and the structure of genetic correlations across environments depend on the genetic architecture of phenotypic plasticity. Although it is likely that multiple mechanisms play a role in the evolution of plasticity, a readily testable hypothesis is that rapid evolution of phenotypic plasticity is, at least initially, more likely to involve genetic variation in transcriptional regulation.

In addition to this evolutionary context, there is increasing interest in a variety of fields as to how environmental factors such as nutrition or exposure to stress influence a wide variety of health-related outcomes such as aging (Gems and Partridge 2013). Although the direct negative effects of some environments, such as exposure to pathogens, are clear, in some cases brief exposure to stress at one point in the life cycle can lead to increased resistance to stress at a later time period—a phenomenon known as hormesis (Gems and Partridge 2008; Le Bourg 2009). In general, it appears that protection via a hormetic response is generated by the induction of stress response pathways (*e.g.*, heat shock proteins) (Volovik *et al.* 2012) in advance of when they are actually needed during exposure to a more severe stressor. Hormesis is a classic example of adaptive phenotypic plasticity, although the intellectual traditions of the two fields are largely distinct.

Here, we address this broad set of evolutionary and functional questions using experimental evolution in nematodes to investigate changes in phenotypic plasticity for an ecologically relevant trait: resistance to heat stress. Experimental evolution has proven to be a powerful system for studying evolutionary processes (Rose *et al.* 1990; Huey *et al.* 1991; Lenski *et al.* 1991; Matsumura 1996; Callahan 2005), including the genetic assimilation of phenotypically plastic traits (Waddington 1953, 1956; Suzuki and Nijhout 2006). Experimental evolution is particularly useful when ancestral and evolved populations can be compared simultaneously (Lenski *et al.* 1991).

We evolved the nematode *Caenorhabditis remanei*, which, like its sister species *C. elegans*, can be frozen indefinitely and recovered later (Brenner 1974). Unlike *C. elegans*, however, *C. remanei* populations display an abundance of genetic variation (Graustein *et al.* 2002; Jovelin *et al.* 2003; Cutter *et al.* 2006) and ample recombination because of obligate outcrossing, both of which facilitate a rapid response to selection (Morran *et al.* 2009). We evolved lines by selecting on their ability to withstand heat shock during early larval development, a trait that displays significant heritable variation in natural populations of *C. remanei* (Reynolds and Phillips 2013). Plasticity for heat stress resistance was measured in populations that were raised in the selective conditions (standard laboratory environment at 20°) and in a high-temperature environment at 30°, which the populations had not experienced during selection. We further investigated the transcriptional changes occurring in the selective populations across environments. Together, these data enable a detailed investigation of adaptive physiological and transcriptional changes in phenotypic plasticity in an ecologically important trait in *C. remanei*.

## Materials and Methods

### Creation of ancestral population

To obtain a heterogeneous population, we collected wild isolates of *C. remanei*. Two hundred woodlice (terrestrial isopods of the Family *Oniscidea*, also known as sowbugs or pillbugs) from Koffler Scientific Reserve at Jokers Hill, King City, Toronto, Ontario (+44° 1′ 46.88′′, −79° 31′ 41.69′′) were graciously provided to us by the Cutter laboratory (University of Toronto) and express-mailed to the Phillips laboratory (University of Oregon). All woodlice were collected within 300 meters of the main building of the field station. Of the 200 woodlice, approximately 20% contained *C. remanei*. From each of these we collected and maintained one mating pair, yielding 26 “isofemale strains.” Isofemale populations were immediately expanded to a large population size following the initial mating (approximately 100–1000 offspring per line in the first generation and very large population sizes in subsequent generations). All collected strains were frozen within three generations of collection to minimize laboratory adaptation. To create a cohort representative of naturally segregating variation for experimental evolution, we thawed samples from each of the 26 isofemale strains and crossed them in a controlled fashion to promote equal contributions from all strains, including from mitochondrial genomes and X chromosomes. The resulting genetically heterogeneous population (PX443) was frozen after creation and served as the ancestral population for the experimental evolution. Polymorphism in this species is ∼5% (Jovelin *et al.* 2003; Cutter *et al.* 2006; Jovelin *et al.* 2009), so there should have been abundant segregating variation present at the initiation of selection. All natural isolates, as well as the lines used in the experiment described below, were grown on nematode growth media (NGM) seeded with *E. coli* strain OP50 (Brenner 1974).

### Stress response phenotype

An acute stress in the context of this experiment is one that challenges the stress response of the worm within a 4-hr period. Given the short average lifespan of *Caenorhabditids* (∼20 d from L4), we reasoned that any exposure in excess of 6 hr might be treated by the worm as a chronic stress and could potentially invoke a fundamentally different class of cellular stress response. To test resistance to acute heat stress, worms were stage-synchronized via a bleaching procedure (“hatch off”) that kills adults and leaves only developing embryos. Embryos were rinsed, suspended in buffer without food, and given 18 to 24 hr to develop into L1 larvae. L1 larvae enter developmental arrest in the absence of food (Baugh 2013). Worms in L1 diapause suspended in liquid buffer were then exposed to an acute heat stress at 36.8° in a shaking incubator (70 rpm) for 4 hr in a sealed microcentrifuge tube. As a control, a subset of the population was kept at 20° under similar conditions. After acute stress shock, worms were transferred into a Petri dish containing Nematode Growth Medium-lite (NGM-lite; U.S. Biological) seeded with *E. coli* strain OP50. Survival was estimated 3 to 4 d later, when most worms had developed into fourth-stage L4 larvae but had yet to lay eggs. Acute heat shock resistance was quantified as the proportion of the phenotyped population that survived the heat shock and matured to adulthood, relative to the average survival of the control samples.

### Experimental evolution

We propagated four laboratory-adaptation control replicates and two acute heat-selected replicates. Each replicate population comprised 1000 to 2000 mating individuals. Exposure to acute stress occurred either every second generation or when the population produced ≥24,000 eggs, whichever occurred later. At that point, worms were stage-synchronized as described previously and subjected to the stress phenotyping protocols as described above. The control populations were randomly culled to 1000 L1 larvae during each selective generation and subjected to similar treatment as the heat shock lines, but at 20°. In the heat-selected populations, 10,000 of the L1 worms were randomly selected to undergo acute stress selection at an average temperature of 36.8°. This intensity of heat shock induces ∼70% larval death in the ancestral population (*s* = 0.7). To maintain a similar strength of selection (*s* between 0.7 and 0.8) throughout the experiment as the heat-selected population adapted, the heat shock temperature was increased incrementally (up to 37.2° in the final generation of selection). The populations were maintained in standard laboratory conditions at 20° between selective events. Selection was continued until each replicate line had experienced 10 total selective events.

Each population was frozen (*N* ≥ 100,000 individuals) after approximately every second generation of experiencing acute stress shock to retain a record of evolutionary change in the populations over time and to ensure that worms did not lose the ability to survive freeze and thaw. Approximately 5000 individuals from the frozen populations were thawed to continue the evolution experiment, whereas the remaining 95,000 worms remained frozen for future phenotyping and genetic and genomic analyses. Populations were thawed for selection after a minimum of 24 hr at −80°. In half of the selection lines (two control and one heat-selected population), freezing occurred a total of three times during selection, whereas this occurred five times in the remaining populations.

### Phenotypic plasticity across environments

To measure phenotypic plasticity for heat shock resistance across environments, the parents of phenotyped individuals were reared in either typical laboratory conditions (20°) or mild heat stress (30°). Their offspring were stage-synchronized, grown, and phenotyped in the parental rearing environment, *e.g.*, worms whose parents experienced 30° were raised entirely at 30°. The heat shock assays were performed as described above, except that the control samples were also kept at the same rearing temperature during the phenotyping assay.

Additionally, we chose the ancestor and one representative population from each selective regime to measure resistance to heat shock at a range of temperatures from 36.5° to 37.8°. The temperature during heat shock was recorded at 5-min intervals using two Thermochron iButton devices (Maxim Integrated). The average heat shock temperature was defined as the average measured temperature for both devices over 4 hr. Heat stress across the range of heat shock temperatures was measured as described for the standard (36.8°) heat resistance assays.

### Statistical analysis of phenotyping data

We tested for differences in survival following heat shock using a mixed model ANCOVA using JMP10.0 (SAS Institute). Fixed effects in the model included the cultivation temperature for phenotyping (20° or 30°), the selection regime (ancestor, control-selected, or heat-selected), and the interaction between phenotyping temperature and selection regime. Independently derived replicate lines from each experimental block were nested within selection regime and treated as a random factor using Satterthwaite’s approximation for degrees of freedom (Winer *et al.* 1991). As the dependent variable, we used square-root transformed counts of survivors from each heat-shocked plate. We included the square-root transformed average count of worms from the control plates for each phenotyping assay as a covariate in the model to control for variation in the estimated number of worms in each assay. The interaction between replicates and phenotyping environment was also included in the full model as a random factor, but its effect was very small and not significant and produced a slightly negative variance component estimate. Therefore, we set the variance component equal to zero for this term to perform further hypothesis testing.

Differences between reaction norms over the range of heat shock temperatures were tested by fitting a logistic regression model implemented in R (R Development Core Team 2013). We used a quasi-binomial model to allow for overdispersion in the response variable. The total number of individuals in each trial was assumed to be the average count from controls from the same treatment group that were assayed concurrently. The number of survivors from each heat shock trial was taken to be the successes in the model. In any case in which the number of survivors was greater than the assumed total, the number of survivors was assumed to be equal to the total (100% survival). Two factors, rearing environment and selection regime, as well as one continuous variable, average heat shock temperature, and all interactions were tested in the full model. We also tested for environment and environment-by-heat shock temperature interactions within each selection line.

### Transcriptional profiling of pooled populations

To obtain tissue for transcriptional profiling experiments, we thawed frozen stocks of worms from the ancestral population, one representative control population, and one heat-selected population. Worms were raised at 20° for a minimum of three generations or until the population was at least 250,000 individuals. Each population was then allowed to lay eggs, which were age-synchronized as described above. Age-synchronized embryos were allowed to hatch and develop for 20 hr in liquid medium, at which time most individuals had entered L1 diapause. Half of the larvae developed at 20° during this period, which we define as the larval development environment, whereas the remainder developed at 30°. After 20 hr, larval worms were passed through a 20-μm Nitex screen to remove unhatched eggs and dead adults. Approximately 15 μl of pelleted L1 tissue (∼100,000 individuals) was flash-frozen in TRIzol (Ambion) and stored at −80° until RNA isolation. For each treatment condition from each line, six replicates were collected from a minimum of two independently thawed populations from each line. We extracted total RNA from L1 tissue using standard TRIzol methods, and from this pool mRNA was isolated using the Dynabeads mRNA purification kit (Ambion). Purified mRNA was sheared to 200- to 600-nt fragments using a buffered zinc solution (RNA Fragmentation Reagent; Ambion). cDNA was synthesized using Superscript III reverse-transcriptase (Invitrogen), and sequencing libraries were created through ligation of adaptors with inline barcodes to enable multiplexing of samples. Samples were sequenced in five lanes on an Illumina HiSequation 2000 at the University of Oregon Genomics Core Facility.

### Analysis of differential gene expression

We performed quality filtering of raw sequence reads using the process_shortreads component of the software Stacks (Catchen *et al.* 2011, 2013), which discards reads with ambiguous sample identity, reads with uncalled bases, and reads failing Illumina purity filters. Reads with ambiguous barcodes were rescued if they had fewer than two mismatches from a known barcode. We obtained more than 342 million reads that passed initial quality filters. We aligned all reads that passed the quality filters to the *C. remanei* genome (C_remanei-15.0.1 assembly) available at Ensembl Metazoa (metazoa.ensembl.org/‎) using GSNAP (Wu and Nacu 2010). To help guide the alignment across exon boundaries, we used existing annotated gene models for protein-coding genes obtained from Ensembl Metazoa while allowing GSNAP to identify novel splice sites as well. For this study, we chose to focus on previously annotated protein-coding genes. While this approach may miss responses in genes that are not currently annotated, this dataset does include 31,518 transcripts, including many of the genes that might be expected to respond to heat stress (*e.g.*, hsps). We then used the htseq-count tool from the Python package HTSeq (http://www-huber.embl.de/users/anders/HTSeq/) to count all reads aligning to protein-coding genes. Reads were counted against the gene models using the “union” mode in htseq-count, so that reads were only counted if they unambiguously overlapped a single gene model.

For each selection line, we tested only those genes for which we could confidently detect expression. Genes expressed at very low levels are unlikely to be detected in all libraries and are more likely to be affected by sampling variance (Bloom *et al.* 2009), thereby reducing the power for detecting differential expression among treatments. We modified the filtering procedure commonly implemented in the edgeR package (Robinson and Oshlack 2010; Anders *et al.* 2013) to remove these uninformative genes prior to analysis. Genes that had less than one count per million (cpm) were considered to be unexpressed in a given sample. In our smallest sequenced libraries, 1 cpm is equivalent to approximately two reads aligned to a given gene. Because we were interested primarily in the effect of environmental treatment, we excluded genes for analysis unless they met the detection threshold (>1 cpm) in at least four of the six replicates for one of the temperature treatments for any line.

Differential gene expression analysis was conducted using the DESeq package (Anders and Huber 2010) in R, which utilizes a negative binomial distribution to test for differential expression among treatments to better accommodate the well known phenomenon of overdispersion in RNA-seq data. We tested for differences in gene expression between larval environments within each of the selection lines. Two factors, larval development temperature and replicate thaw, were included in the full model as additive effects. To assess the effect of temperature on expression, we compared the full model to a reduced model that excluded temperature. Larval development temperature was deemed to have a significant effect on the regulation of a gene if the full model fit significantly better than the reduced model at a 5% false discovery rate (FDR) after adjusting for multiple comparisons using the Benjamini-Hochberg method (Benjamini and Hochberg 1995). Similarly, we tested for the effect of evolution within the 20° larval environment by testing for significant expression differences between each pair of populations.

To understand how transcriptional plasticity evolved in the selected lines, we compared differential expression (log_2_ fold change between larval environments) of each evolved population to the ancestor. Because of differences in power to detect differential expression among the three lines, we used a regression approach to compare the average change across environments in each selected line and the ancestor. Three genes that were expressed in only one environment were excluded from this regression analysis, because the log_2_ fold change is infinite. Furthermore, we excluded from this analysis all genes that were expressed below the detection limit in either compared line, as gene silencing potentially represents a different mechanism for genetic assimilation. Finally, we also excluded genes that did not show significant inducible expression (FDR <5%) in at least one of the two lines undergoing comparison. We fit an ordinary least squares (OLS) linear model (using the lm function in R) to the log_2_ transformed fold change of the significantly differentially expressed genes for each pairwise comparison of the evolved lines with the ancestor.

### Gene ontology enrichment analysis

We used the software program Blast2GO (Conesa *et al.* 2005) to look for over-representation of GO terms (The Gene Ontology Consortium 2000) in the sets of significantly upregulated or downregulated genes in the 30° larval environment. Blast2GO computes a Fisher’s exact test with a FDR correction to test for significant over-representation of GO terms in a test set. Two test sets were created for each population: one with significantly upregulated genes (FDR < 1%) and one with downregulated genes (FDR <1%). We tested for over-representation of generic GOSlim ontology terms using a one-tailed test against a reference set of the genes that were not differentially expressed between larval environments in the same population (FDR >5%). Ontology information was visualized using Cytoscape 3.0 (Smoot *et al.* 2011).

## Results

### Selection increases resistance to heat stress in the selective environment

**Figure 1:**
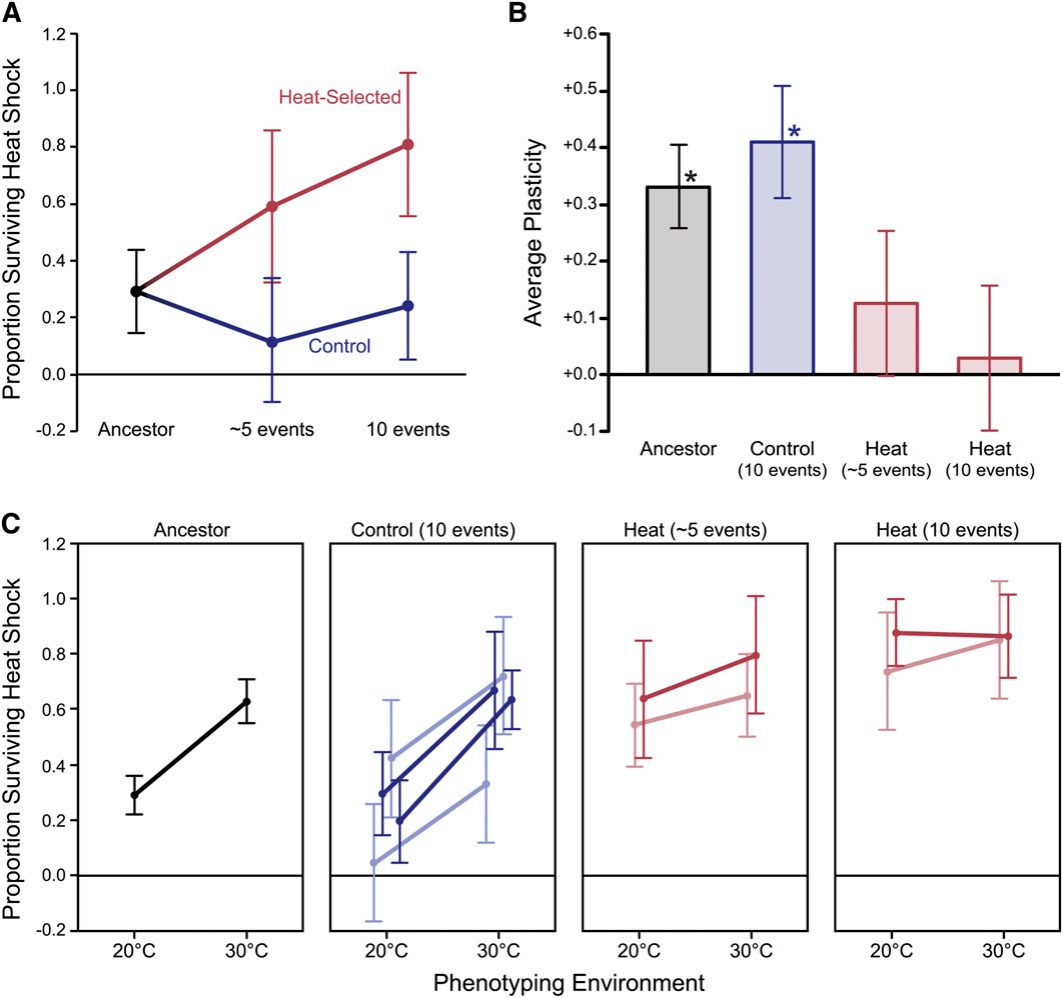
Evolved changes in heat shock resistance in selected lines of *C. remanei*. (A) Proportion of heat-shocked worms surviving to adulthood relative to control treated replicates for populations subjected to heat selection (red) and control populations (blue). (B) Plasticity for heat stress resistance, defined as the difference between survival at 30° and survival at 20° for ancestral (gray), control (blue), and heat-evolved (red) populations. Asterisks denote populations with a significant (*P* < 0.05) effect of environment (*i.e.*, plasticity) on survival. (C) Reaction norms for replicate evolved lines in the 20° and 30° environments. Least squared means from the ANOVA with 95% confidence intervals are plotted.

When raised in standard laboratory conditions at 20°, approximately 30% of individuals from the ancestral population survived a 36.8° heat shock treatment during the early larval period and were subsequently able to develop to adulthood (Figure 1A). After ∼30 generations of propagation under standard laboratory conditions, control populations maintained a level of heat stress resistance that was approximately comparable to that of the ancestor (F_1,4_ = 0.99, *P* = 0.3825). Some variation among independently evolved replicates was observed, potentially reflecting genetic drift among these populations. In contrast, selection via periodic exposure to heat shock increased resistance to high temperatures. Comparing the time points from the heat-selected lines reveals a linear increase in survival over the course of selection (linear model with time: F_1,4_ = 10.04, *P* = 0.0397; quadratic terms: F_1,5_ = 0.23, *P* = 0.6485), culminating with nearly 85% of individuals surviving heat shock in the final generation.

### Plasticity for acute heat shock resistance evolves rapidly

In addition to measuring larval heat resistance after cultivation at 20°, which is the standard environment during the selection experiment, we also exposed individuals from each population to a novel environment: elevated temperature (30°) during embryogenesis. Note that *C. remanei* is much more resistant to high temperatures than *C. elegans*, which tends to have an upper thermal limit of 26° to 27° (Anderson *et al.* 2011). After cultivation at 30°, survival of the ancestral population increased to 63% after heat shock (F_1, 111_ = 33.52, *P* < 0.0001) (Figure 1C), reflecting a high degree of plasticity across environments for the heat resistance phenotype (Figure 1B), apparently via induction of heat resistance pathways at this sublethal temperature. In the populations evolved under control conditions, the novel 30° environment induced a similarly large plastic response as in the ancestral population, with no significant population-by-environment interaction (F_1, 113_ = 2.82, *P* = 0.0959) (Figure 1B). Despite some variation in average survival at each cultivation temperature, plasticity in survival was highly consistent across all controls (Figure 1C).

In lines selected for heat shock resistance, there was no significant increase in survival in the novel environment at 30° compared to the ancestor (F_1, 4_ = 1.84, *P* = 0.2462), which contrasts sharply with the response observed at 20°. Consequently, plasticity across environments declined dramatically during selection until the complete loss of environmental sensitivity occurred after 10 generations of selection for heat resistance (F_1, 112_ = 1.11, *P* = 0.6558). Loss of plasticity occurred in this case because the phenotype in the 20° environment evolved to match that of the 30° environment. Note that this result is not simply a matter of scale, as these populations were still relatively far from the upper bound of 100% survival. These results support an apparent genetic assimilation of the heat-induced phenotype following selection.

### Global transcriptional response to environmental change is unchanged

**Figure 2:**
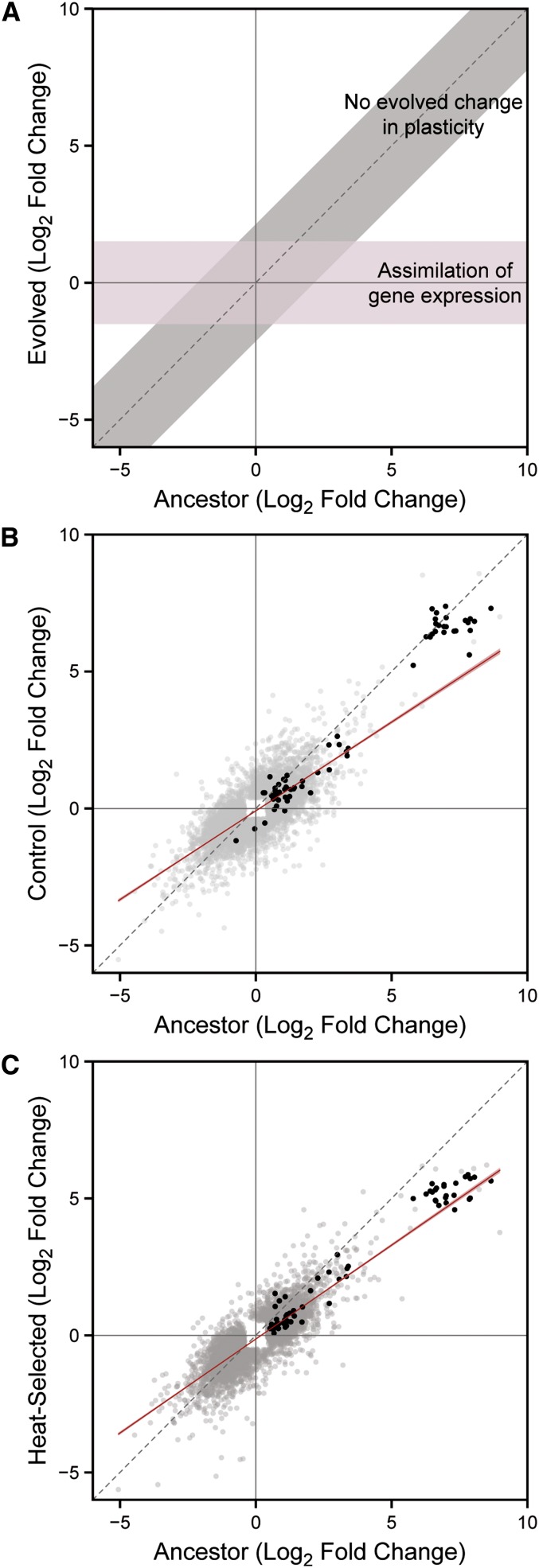
Inducible transcriptional response in evolved lines of *C. remanei*. (A) Predicted changes in transcriptional plasticity between the ancestor and heat-evolved populations under a null hypothesis in which genes have equivalent levels of plasticity in both selection lines (*i.e.*, no change in plasticity) or the alternative hypothesis in which there is genetic assimilation of gene expression in the evolved line. (B) Comparison of changes in inducibility of gene expression at 30° in ancestor *vs.* the control evolved populations or (C) ancestor *vs.* heat-selected. Gray points represent differentially expressed genes from either of the compared lines, and black points represent candidate hsp genes. Red lines in (B) and (C) indicate the linear fit from the regression model (±95% CI). Gray dashed line is slope of 1, representing the null hypothesis of equal expression between lines.

Given the observed pattern of rapidly evolved plasticity for heat stress resistance across environments, we hypothesized that the phenotypic evolution may be manifested in differences in gene expression profiles across environments in a large proportion of genes. Furthermore, we predicted that the phenotypic assimilation might be matched by a pattern of transcriptional assimilation as well. Specifically, genes that are differentially expressed between the 20° and 30° environments in the ancestral population would be expected to become constitutively expressed in the heat-evolved lines to match the observed phenotypic change in those populations (Figure 2). To test this hypothesis, we used RNA-sequencing (henceforth RNA-seq) on pooled samples from the ancestor, control, and heat-evolved populations, which were divided and raised at either 20° or 30° for 20 hr before tissue collection.

Of the genes that were expressed above our threshold for detection, we identified 8377 genes that were differentially expressed across environments in at least one of the three populations (Supporting Information, Table S1). Not surprisingly, exposure to the 30° environment caused upregulation of genes involved in mediating response to stress (Figure S1 and Table S2), as well as enrichment of biological processes related to metabolism, growth, and development. Processes relating to ion transport and cellular communication were downregulated at 30°. These processes were similarly enriched in all three selection lines.

To understand how expression plasticity has evolved in the selected populations, we compared the inducibility (measured as the log_2_ fold change in expression between environments) of the differentially expressed genes between selection lines and the ancestor (Figure 2). When comparing the expression change across environments between different lines, the null expectation is that these should have equal expression differences in both lines for the majority of genes (Figure 2A). Alternatively, under a genetic assimilation model, we would expect to observe expression differences in the control and ancestral populations but constitutive expression of these genes in the heat-evolved population.

When comparing the expression change in the ancestral population to the expression difference in either evolved population, there was a slight reduction in the slope term of the linear model, suggesting that many genes are somewhat less responsive to environmental change (Figure 2, B and C). However, this effect was apparent in both the control (*b* = 0.648; *t*_7751_ = −59.49; *P* < 0.0001) and heat-evolved populations (*b* = 0.686; *t*_6743_ = −60.63; *P* < 0.0001), indicating that this observed pattern may be a signature of laboratory adaptation rather than genetic assimilation of heat stress resistance. The reduction of slope was slightly more pronounced in the control populations than in those selected for heat resistance (*t*_14494_ = 4.78, *P* < 0.0001). Furthermore, a significant correlation between the responsiveness of expression in the ancestor and evolved populations remains, implying that general transcriptional assimilation is not responsible for the phenotypic assimilation.

### Inducibility of candidate heat shock proteins is unchanged

Genetic assimilation may not be generated by constitutive gene expression at a global level, but rather by changes in specific pathways such as those regulating heat response. To test this hypothesis we analyzed the response of heat shock proteins, which are particularly strong candidates for regulating a heat-specific response because of their key role in mitigating damage due to cellular stress (Lindquist and Craig 1988). In addition, *hsp70* genes have been shown to respond to selection at different temperatures in *Drosophila melanogaster* (Bettencourt *et al.* 2002), and the inducibility of *hsp70* differs among related *Drosophila* species adapted to different thermal environments (Krebs 1999; Calabria *et al.* 2012), making these genes likely targets for adaptation to heat stress. We identified 89 genes in *C. remanei* belonging to four families of heat shock proteins: the HSP70 superfamily; the HSPC (HSP90) family; the DNAJ (HSP40) family; and small heat shock proteins in the HSPB family, many of which are inducible in response to heat stress in the genus *Caenorhabditis* (Heschl and Baillie 1990; Stringham *et al.* 1992; Nikolaidis and Nei 2003). As expected, most of the hsps exhibited a high degree of plasticity across environments. However, they also retained an equivalent degree of plasticity in both evolved populations (Figure 2). In addition, there was no evidence that the basal level of hsp expression at 20° differed among populations. A few genes did show altered expression over evolutionary time (Table S3), but there was no clear pattern of constitutive upregulation across stress response pathways. Thus, despite their canonical role in mediating heat shock response, hsps do not appear to play a role here in the apparent genetic assimilation of heat shock resistance in the selected population.

### Genetic assimilation of heat resistance is only apparent and is context-dependent

**Figure 3:**
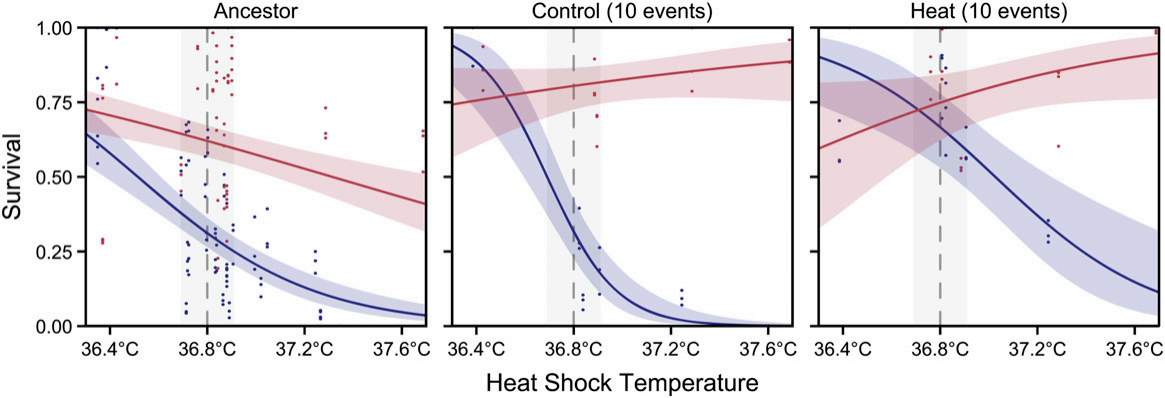
Evolved changes in heat shock reaction norm in evolved lines of *C. remanei* occur by shifting the reaction norm. Shown are predicted reaction norms across a range of heat shock temperatures for a representative of each selection line raised at either 20° (blue) or 30° (red). Points represent the proportion of worms surviving in replicate trials, and solid lines indicate the predicted probability of survival from a logistic regression with 95% confidence intervals. The gray box indicates the temperature range included in the 36.8° heat shock assays, where plasticity was initially measured.

Given the discordance in evidence for assimilation at physiological and transcriptional levels, we sought to understand whether the transcriptional response to temperature might underlie a more complex relationship between the environment and phenotype by exploring the evolved norms of reaction for survivorship over a broader range of heat shock temperatures. As in the single temperature assays, we observed a significant interaction between heat shock temperature, population, and rearing environment (F_2, 240_ = 8.17, *P* = 0.0004), indicating that evolved differences in plasticity due to rearing environment affect the rate of survival across the range of heat shock temperatures (Figure 3). In particular, as above, rearing environment had strong effects on resistance within the ancestor (rearing environment-by-heat shock temperature interaction: F_1, 176_ = 18.95, *P* < 0.0001) and control lines (interaction: F_1, 176_ = 53.17, *P* < 0.0001). However, rearing the worms at 30° also induced increased heat shock resistance in the heat-selected lines when the heat shock occurred at temperatures above the selection temperature (>37°; interaction F_1, 35_ = 20.75, *P* < 0.0001). In fact, the heat-selected populations appear to have evolved greater plasticity at high heat shock temperatures, largely by improving inducible heat shock resistance after being raised at 30°. Thus, while assimilation is evident at the specific temperature utilized under direct selection, plasticity is maintained—and even enhanced—at a broader spectrum of formally lethal temperatures.

## Discussion

Organisms live in a constantly varying world. In response to this environmental variation, numerous lineages have evolved the ability for individuals to predictably modify their phenotypes in response to environmental heterogeneity. The importance of phenotypic plasticity in influencing ecological and evolutionary processes—such as modifying the probability of extinction or influencing the trajectory of evolutionary response—has long been known by biologists (Baldwin 1896; Morgan 1896; Waddington 1942; Schmalhausen 1949; Bradshaw 1965; West-Eberhard 2003). Despite this recognition, studies of the origin and evolution of phenotypic plasticity, in particular how quickly it can evolve and the genetic basis of plasticity, have been unsatisfactorily inconclusive. One reason is that labile phenotypes that vary in response to environmental change could have naively been seen as lacking any genetic basis and therefore unable to evolve. However, evolutionary biologists now correctly understand that the ability to coherently respond to environmental variation is itself a trait that can evolve and that genetic variation for this trait can be sorted within and among populations (Via 1984). A more important reason for the lack of progress is the difficulty of using comparative studies of phenotypic plasticity in evolved populations to directly address questions of evolutionary rate and genetic mechanism. In this study, we have tackled these holes in our understanding of the evolution of phenotypic plasticity by using a powerful experimental evolution approach.

One specific form of plasticity of broad interest to molecular as well as evolutionary biologists is the increased hardiness that can often be induced by low doses of a toxin or brief exposure to a stressful environment (Calabrese and Baldwin 2003). The induced response, or hormesis, is presumably caused by the upregulation of stress-resistance factors in the initial exposure that then serve a protective function in subsequent, and potentially harsher, exposures (Gems and Partridge 2008). Within *C. elegans*, brief exposure to high temperatures has been shown to yield increased resistance to high temperatures (Lithgow *et al.* 1995) as well as increases in longevity (Gems and Partridge 2008; Le Bourg 2009). Hormesis is not usually discussed in the context of phenotypic plasticity—although it is precisely that—and in this case serves as example of adaptive phenotypic plasticity. There is evidence for genotype-by-environment interaction for this response in *C. elegans* (Rodriguez *et al.* 2012), which is a necessary precursor to the evolution of a plastic response. Here, we find an essentially similar (if not stronger) hormetic response in populations of the closely related and genetically diverse nematode *C. remanei* that have been recently collected from nature. Thus, this pattern of plasticity appears to be highly conserved across this group of nematodes.

Despite this conservation, selection for resistance to nearly lethal high temperatures rapidly produced a complete loss of plasticity for resistance to heat stress in independently evolved replicate lines. Dramatically increased fitness and a complete loss of plasticity were observed after only 10 generations of selection (Figure 1). This pattern of genetic assimilation was very similar to that predicted by Waddington (1953; 1956) more than 60 years ago, but it occurred much more quickly than what may have been otherwise expected. The tempo of this plasticity change could only be assayed in an experimental evolution framework and led to an important subsequent question: how could such rapid evolution occur? Changes in the frequencies of alleles that affect coding sequences of genes or alleles of regulatory elements affecting the levels of expression of different genes could be responsible. We addressed the latter hypothesis and found that the global patterns of gene expression have not been altered in a way that matches the genetic assimilation of the phenotype (Figure 2).

In contrast to the expectation of global genetic assimilation in transcription, a more focused hypothesis is that particular candidate pathways would experience genetic assimilation. For example, given what is known about the genetics of heat shock resistance (Lindquist and Craig 1988; Morimoto 1998; Volovik *et al.* 2012), one simple means of achieving this pattern of assimilation would be the constitutive upregulation of heat shock protein genes at permissive temperatures, thereby allowing these proteins to provide ready-made protection without the need for them to be induced before rapid exposure to lethal temperatures. Surprisingly, we also did not observe the predicted changes in gene expression levels in these key proteins. Instead, most of the hsp genes that showed differential expression in one or more selection lines showed a high degree of correlation in expression across treatments, and most of the decrease in the environmental induction of expression seemed to result from laboratory adaptation rather than specific assimilation in the heat-selected line. Thus, neither global nor hsp-focused gene expression patterns evolve in a pattern consistent with the genetic assimilation of the phenotype.

There are several explanations for the divergent observations of genetic assimilation at the level of the phenotype but concurrent lack thereof at the level of gene expression. First, the phenotypic response may be a result of changes in a few key stress response genes. However, the strongest candidates for regulating the heat shock response, the hsps, respond similarly in all lines. A second possibility is that the basal level of gene expression among lines is more important in the heat-selected line, and additional induction of expression under heat stress does not further improve survival. A few genes may be in this category and require further study (Table S3). Alternatively, constitutive upregulation may be important, but the target of regulation (*e.g.*, protein degradation or post-translational modification) might not be revealed from an analysis of transcript levels.

In contrast to these strictly genetic explanations, another possibility is that our initial finding of phenotypic assimilation is only apparent (Figure 3). In addition to the shift of the reaction norm when raised at 20°, there appears to be a correlated shift in the 30° reaction norm as well, so that plasticity is actually increased at temperatures beyond those initially assayed. This indicates that strong genetic correlations for heat resistance exist between the stressful and permissive environments, as predicted by Via and Lande (1985), and that such correlations may strongly influence the phenotype across multiple environments. Thus, genetic assimilation of the heat resistance response was apparent only and limited to a narrow window of possible environmental perturbations.

It has been long recognized that the specifics of phenotypically plastic responses are dependent on the exact environments in which they are measured (Bradshaw 1965). For example, Waddington saw genetic assimilation as a specific form of canalization, or reduction in phenotypic variation, and hypothesized that canalization could be broken outside the range of environmental variation under which assimilation occurred (Waddington 1942). Our results clearly support this point of view. Even in the context of a significantly reduced set of environmental stimuli, as examined here, it is apparent that the phenotypic and environmental space is complex and multidimensional. Although the evolution of genetic assimilation might be seen as potentially limiting subsequent evolutionary change, traits that presumably exhibit canalization in one range of environmental variation are likely to be periodically exposed to ranges of environmental conditions under which canalization is broken. Therefore, rather than limiting the evolutionary response to selection via the induction of genetic canalization, changing environments instead likely provide a continually shifting substrate for the evolution of plasticity. The dynamic balance between canalization and plasticity is therefore one of the major drivers—and outcomes—of evolution in a complex environmental milieu.

## Supplementary Material

Supporting Information
