# Supplementary material for: Rapid Evolution of Phenotypic Plasticity and Shifting Thresholds of Genetic Assimilation in the Nematode Caenorhabditis remanei
Source: G3 (Bethesda). 2014 Apr 11;4(6):1103–12. doi: 10.1534/g3.114.010553 (PMC4065253; doi:10.1534/g3.114.010553)
Supplement: Supporting Information [file supp_4_6_1103__index.html]

Rapid Evolution of Phenotypic Plasticity and Shifting Thresholds of Genetic Assimilation in the Nematode Caenorhabditis remanei — Supporting Information 

# Rapid Evolution of Phenotypic Plasticity and Shifting Thresholds of Genetic Assimilation in the Nematode *Caenorhabditis remanei*

## Supporting Information for Sikkink *et al.*, 2014

**Files in this Data Supplement:**

- Supporting Information - Figure S1, Tables S1-S3, and Files S1-S2 (PDF, 430 KB)
- Figure S1 - Gene ontology enrichment network for genes differentially expressed by environment. (PDF, 91 KB)
- Table S1 - Summary of differential expression results by line. (PDF, 39 KB)
- Table S2 - List of the 200 most differentially expressed genes across environments from the RNA-seq analysis. (PDF, 139 KB)
- Table S3 - List of genes differentially expressed over evolutionary time (20ºC environment). (PDF, 81 KB)
- File S1 - Phenotypic responses for the experimental evolution lines. (.csv, 9 KB)
- File S2 - Data for the full heat shock reaction norm for each line. (.csv, 14 KB)
