# Supplementary material for: Rapid Evolution of Phenotypic Plasticity and Shifting Thresholds of Genetic Assimilation in the Nematode Caenorhabditis remanei
Source: G3 (Bethesda). 2014 Apr 11;4(6):1103–12. doi: 10.1534/g3.114.010553 (PMC4065253; doi:10.1534/g3.114.010553)
Supplement: Supporting Information [file supp_g3.114.010553_010553SI.pdf]

G3: Genes | Genomes | Genetics

Rapid evolution of phenotypic plasticity and shifting thresholds of genetic assimilation in the nematode *Caenorhabditis remanei*

Kristin L. Sikkink*, Rose M. Reynolds*,†, Catherine M. Ituarte*, William A. Cresko*[1], and Patrick C. Phillips*[1]

*Institute of Ecology and Evolution, University of Oregon, Eugene, Oregon 97403-5289, †Department of Biology, William Jewell College, Liberty, Missouri 64068

RNA-seq data are deposited in the NCBI Gene Expression Omnibus (GEO) database as part of series GSE56510 with accession numbers GSM1362987–1363022.

[1]Corresponding authors: Institute of Ecology and Evolution and Department of Biology, University of Oregon, Eugene, OR 97403. E-mail: wcresko@uoregon.edu, pphil@uoregon.edu.

DOI: 10.1534/g3.114.010553

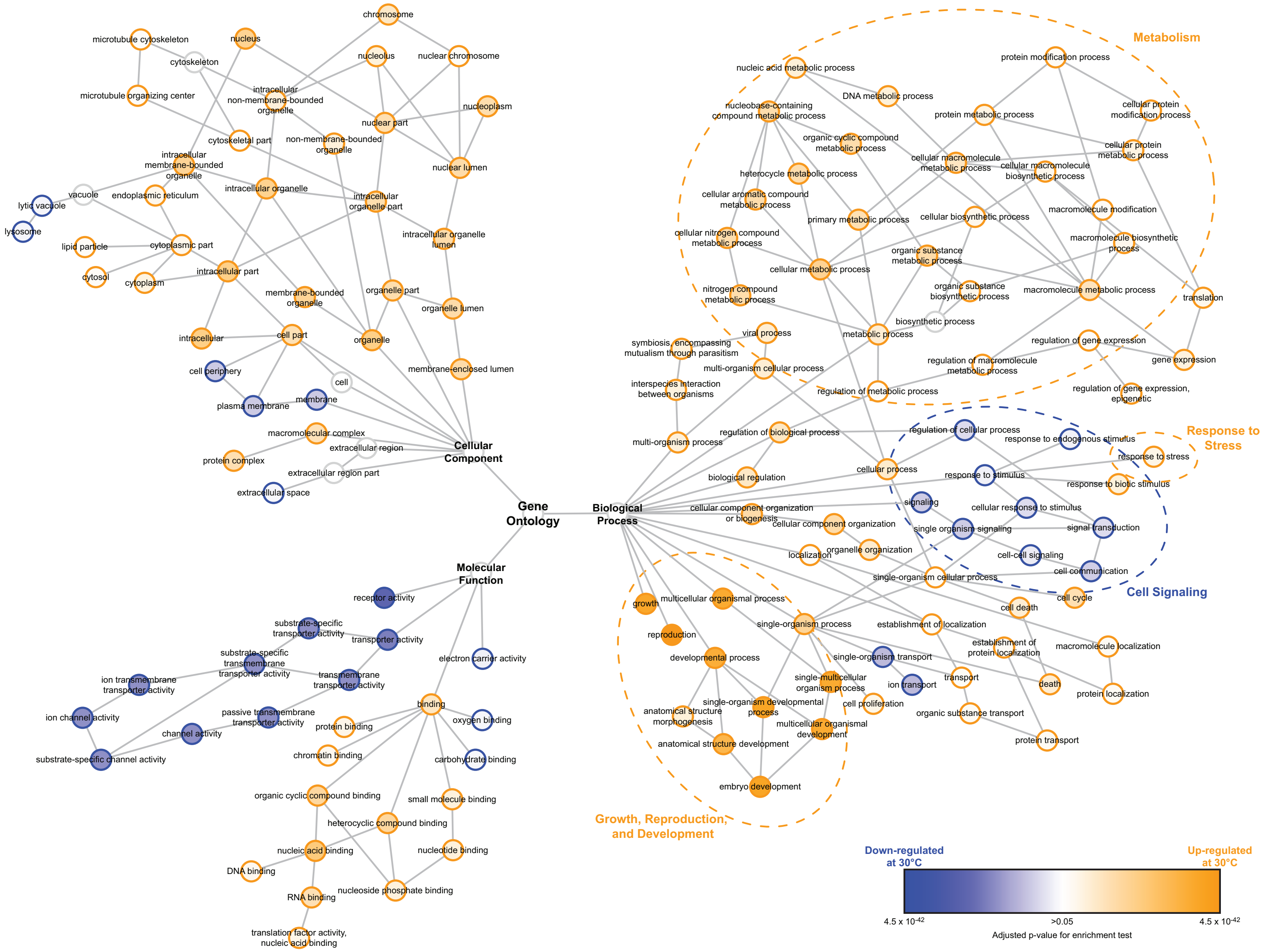

Figure S1 Gene ontology enrichment network for genes differentially expressed by environment. Shown are GOSlim terms with significant enrichment (FDR < 5%) for the up-regulated genes (orange) or down-regulated genes (blue) in the ancestral population. The intensity of shading is proportional to the significance value from the Fisher's Exact Test for the ontology term.

Table S1 Summary of differential expression results by line.

Line	Genes expressed above threshold	Differential expression (FDR 5%)
Ancestor	15,347	6431
Control	15,141	4286
Heat	14,784	2769
Combined (all lines)	15,963	8377

Table S2 List of the 200 most differentially expressed genes across environments from the RNA-seq analysis. Dashes indicate that the gene was not detected above our detection threshold at either temperature for the line under comparison; n.s. indicates that transcript levels were not significantly different between environments.

HSP	Gene ID	Gene Name	Gene Description	Ancestor Log_2 FC	Ancestor FDR	Control Log_2 FC	Control FDR	Heat Log_2 FC	Heat FDR
	CRE09388		C-type lectin	35.31	>0.0001	--	--	4.61	>0.0001
	CRE24573		Protein kinase	-5.31	>0.0001	-34.57	>0.0001	-33.46	>0.0001
	CRE08067			32.35	>0.0001	--	--	--	--
	CRE23800		Hydrolase	8.99	>0.0001	7.00	>0.0001	n.s.	n.s.
◆	CRE24849		HSP70 protein	8.66	>0.0001	7.31	0.0011	5.64	0.0017
	CRE19381			8.22	>0.0001	8.57	0.0024	5.33	0.0344
	CRE18157			6.14	>0.0001	8.52	0.0059	--	--
	CRE18318			8.50	>0.0001	--	--	6.22	0.0198
◆	CRE04868		HSP70 protein	8.05	>0.0001	6.83	0.0116	5.78	0.0006
	CRE26773		Integrase	8.03	>0.0001	6.09	0.0222	6.09	>0.0001
◆	CRE01097		HSP70 protein	7.90	>0.0001	6.50	0.0192	5.73	0.0035
◆	CRE18319		Small hsp (HSPB)	7.90	>0.0001	6.93	0.0035	5.01	0.0002
◆	CRE18317		Small hsp (HSPB)	7.86	>0.0001	5.61	>0.0001	4.97	0.0013
◆	CRE04869		HSP70 protein	7.81	>0.0001	6.79	0.0166	5.87	0.0043
◆	CRE01029		HSP70 protein	7.72	>0.0001	6.86	0.0052	5.79	0.0008
	CRE26772		Reverse transcriptase	7.57	>0.0001	n.s.	n.s.	5.99	>0.0001
◆	CRE19380		Small hsp (HSPB)	6.99	>0.0001	7.38	0.0005	5.03	>0.0001
◆	CRE18323		Small hsp (HSPB)	7.36	>0.0001	6.48	0.0048	5.56	>0.0001
◆	CRE04666		HSP70 protein	7.33	0.0017*	--	--	4.59	0.0188
◆	CRE19334		Small hsp (HSPB)	7.30	>0.0001	6.47	0.0081	5.11	>0.0001
◆	CRE26901		HSP70 protein	6.49	>0.0001	7.28	0.0043	5.54	>0.0001
◆	CRE18316		Small hsp (HSPB)	6.65	>0.0001	7.15	>0.0001	5.38	>0.0001
◆	CRE18322		Small hsp (HSPB)	7.01	>0.0001	6.96	0.0003	5.10	>0.0001
◆	CRE19384		Small hsp (HSPB)	7.01	>0.0001	6.64	0.0017	4.83	>0.0001
	CRE23104			6.95	>0.0001	--	--	--	--
◆	CRE19383		Small hsp (HSPB)	6.93	>0.0001	6.64	0.0006	5.44	>0.0001
◆	CRE25393		HSP70 protein	6.93	>0.0001	6.43	0.0035	5.50	0.0005

HSP	Gene ID	Gene Name	Gene Description	Ancestor Log_2 FC	Ancestor FDR	Control Log_2 FC	Control FDR	Heat Log_2 FC	Heat FDR
◆	CRE19333		Small hsp (HSPB)	6.61	>0.0001	6.91	0.0009	5.31	>0.0001
◆	CRE18315		Small hsp (HSPB)	6.63	>0.0001	6.75	0.0007	4.91	>0.0001
◆	CRE19335		Small hsp (HSPB)	6.74	>0.0001	6.68	>0.0001	4.75	0.0001
	CRE01030			6.62	>0.0001	--	--	6.08	>0.0001
◆	CRE18321		Small hsp (HSPB)	6.61	>0.0001	6.46	0.0004	4.93	>0.0001
◆	CRE27162		Small hsp (HSPB)	6.50	>0.0001	6.37	0.0077	5.23	0.0016
	CRE05591			n.s.	n.s.	-6.47	>0.0001	--	--
◆	CRE20780	*hsp-70*	HSP70 protein	6.42	>0.0001	6.26	0.0003	5.27	>0.0001
	CRE24278		C-type lectin	--	--	--	--	-6.39	>0.0001
◆	CRE27471		Small hsp (HSPB)	6.27	>0.0001	6.27	0.0027	5.16	0.0001
	CRE21296		Reverse transcriptase	6.13	>0.0001	n.s.	n.s.	6.18	>0.0001
	CRE16108		CUB-like domain	--	--	--	--	-6.07	>0.0001
	CRE05459	*end-3*	GATA zinc finger transcription factor	6.05	>0.0001	--	--	--	--
	CRE20711		UDP-glucose:glycoprotein glucosyltransferase	5.87	>0.0001	n.s.	n.s.	4.61	0.0010
◆	CRE19382		Small hsp (HSPB)	5.80	>0.0001	5.22	>0.0001	4.99	0.0018
	CRE27833		Helitron helicase-like domain	5.69	>0.0001	--	--	n.s.	n.s.
	CRE19026		Metridin-like ShK toxin domain	-5.06	>0.0001	-5.51	>0.0001	-5.62	>0.0001
	CRE06466	*sre-42*	Serpentine receptor, class E	-3.61	0.0001	-2.54	0.0028	-5.45	>0.0001
	CRE10142		CUB-like domain	--	--	--	--	-5.39	>0.0001
	CRE11034			5.38	>0.0001	3.81	>0.0001	1.33	>0.0001
	CRE28993		Serpentine receptor, class W	--	--	5.38	>0.0001	--	--
	CRE08692		Serpentine receptor, class Z	--	--	--	--	-5.37	>0.0001
	CRE22721			5.34	>0.0001	--	--	5.10	0.0022
	CRE16387			3.95	>0.0001	3.37	>0.0001	5.24	0.0055
	CRE28585			4.66	>0.0001	n.s.	n.s.	4.19	0.0016
	CRE27404	*gcy-13*	Guanylate cyclase	-1.46	0.0301	--	--	-5.14	0.0015
	CRE24995	*cdh-7*	Cadherin	--	--	4.99	0.0314	--	--
	CRE08101			4.68	>0.0001	--	--	--	--

HSP	Gene ID	Gene Name	Gene Description	Ancestor Log_2 FC	Ancestor FDR	Control Log_2 FC	Control FDR	Heat Log_2 FC	Heat FDR
	CRE09372			3.44	>0.0001	4.64	0.0489	2.87	>0.0001
	CRE20636	*phy-2*	Prolyl 4-hydroxylase	4.52	>0.0001	4.61	>0.0001	2.03	>0.0001
	CRE01098			1.77	>0.0001	n.s.	n.s.	4.59	>0.0001
	CRE10141			--	--	--	--	-4.57	>0.0001
	CRE03576		Hydrolase	3.77	>0.0001	4.55	>0.0001	2.01	>0.0001
	CRE12322			--	--	--	--	4.53	0.0016
	CRE14503		C-type lectin	n.s.	n.s.	-1.38	>0.0001	-4.53	>0.0001
	CRE14636	*clec-140*	C-type lectin	n.s.	n.s.	-1.23	0.0004	-4.51	>0.0001
	CRE09419			4.45	>0.0001	2.64	0.0224	4.50	>0.0001
	CRE08905			-3.46	0.0009	-4.48	>0.0001	--	--
	CRE20697		Hexosyltransferase	n.s.	n.s.	-1.26	0.0003	-4.46	>0.0001
	CRE13476		Thaumatin-like protein	-4.46	>0.0001	-3.03	>0.0001	-3.64	>0.0001
	CRE29499			4.44	>0.0001	--	--	3.55	0.0004
	CRE08033			4.43	>0.0001	--	--	--	--
	CRE23366			4.38	>0.0001	--	--	--	--
	CRE11169			4.36	>0.0001	2.78	0.0011	2.12	0.0411
	CRE24133		Collagen	n.s.	n.s.	-4.36	>0.0001	-2.32	>0.0001
	CRE02480		Nuclear hormone receptor	--	--	-1.70	0.0219	-4.35	0.0001
	CRE16040			2.38	>0.0001	4.28	>0.0001	2.55	>0.0001
	CRE03584			4.24	>0.0001	--	--	3.38	0.0061
	CRE31291			4.22	>0.0001	3.03	0.0043	3.01	0.0053
	CRE24819			4.21	>0.0001	2.84	0.0062	2.04	>0.0001
	CRE01842	*clec-60*	C-type lectin	3.71	>0.0001	4.20	>0.0001	n.s.	n.s.
	CRE07273		Lipocalin-related protein	n.s.	n.s.	4.13	0.0461	1.69	0.0140
	CRE26387			--	--	4.11	0.0054	--	--
	CRE13045		Metridin-like ShK toxin domain	-4.09	>0.0001	-4.10	>0.0001	--	--
	CRE15564		Prolyl 4-hydroxylase	3.97	>0.0001	4.08	>0.0001	2.31	>0.0001
	CRE23551			3.28	>0.0001	--	--	4.07	>0.0001
	CRE06358			--	--	-4.07	>0.0001	--	--

HSP	Gene ID	Gene Name	Gene Description	Ancestor Log_2 FC	Ancestor FDR	Control Log_2 FC	Control FDR	Heat Log_2 FC	Heat FDR
	CRE20525		Serpentine receptor, class E	--	--	--	--	4.07	0.0104
	CRE13953		Fatty acid CoA synthetase family	2.49	>0.0001	2.06	0.0001	4.06	0.0001
	CRE08193			--	--	2.15	0.0266	4.04	0.0130
	CRE21837			2.36	>0.0001	2.52	0.0025	4.01	0.0001
	CRE01268			4.01	>0.0001	1.35	0.0356	2.46	0.0345
	CRE18121	*mlt-10*		4.01	>0.0001	3.68	>0.0001	1.42	>0.0001
	CRE06617		Serpentine receptor, class I	3.98	>0.0001	--	--	--	--
	CRE01964			3.96	>0.0001	3.50	>0.0001	2.23	>0.0001
	CRE01322			3.95	>0.0001	--	--	--	--
	CRE01264			3.95	>0.0001	--	--	--	--
	CRE30275			-2.09	0.0018	-3.88	>0.0001	-2.54	>0.0001
	CRE18513			-3.88	>0.0001	-2.10	>0.0001	-3.19	>0.0001
	CRE13168			--	--	--	--	-3.86	>0.0001
	CRE01279			3.86	>0.0001	1.96	0.0010	n.s.	n.s.
	CRE01306			3.86	>0.0001	n.s.	n.s.	--	--
	CRE22946			-3.84	>0.0001	-2.11	0.0017	--	--
	CRE18158			3.21	>0.0001	n.s.	n.s.	n.s.	n.s.
	CRE08879	*srh-129*	Serpentine receptor, class H	-3.83	0.0003	--	--	-2.50	0.0265
	CRE11248	*srx-85*	Serpentine receptor, class X	3.81	>0.0001	--	--		
	CRE02474			-2.87	>0.0001	-2.74	>0.0001	-3.81	>0.0001
	CRE09165			-3.77	>0.0001	-1.71	0.0006	--	--
	CRE30010	*ech-9*	Enoyl-CoA hydratase	1.91	0.0003	3.76	>0.0001	n.s.	n.s.
	CRE05592			n.s.	n.s.	-3.49	>0.0001	-3.74	>0.0001
	CRE30392			3.73	>0.0001	n.s.	n.s.	n.s.	n.s.
	CRE21032		Flavin monooxygenase	-3.73	>0.0001	-2.79	>0.0001	-1.64	>0.0001
	CRE18358			3.03	>0.0001	--	--	3.73	0.0001
	CRE09421			3.69	>0.0001	3.11	>0.0001	3.11	>0.0001
	CRE03133			-3.69	>0.0001	-1.36	>0.0001	-2.38	0.0226
	CRE17248	*aagr-4*	Acid alpha glucosidase related	2.40	>0.0001	3.68	>0.0001	2.18	>0.0001

HSP	Gene ID	Gene Name	Gene Description	Ancestor Log_2 FC	Ancestor FDR	Control Log_2 FC	Control FDR	Heat Log_2 FC	Heat FDR
	CRE01319			3.68	>0.0001	2.31	0.0023	n.s.	n.s.
	CRE09022			-3.45	>0.0001	-3.66	>0.0001	-2.64	0.0007
	CRE03585			3.65	>0.0001	n.s.	n.s.	3.60	0.0027
	CRE03432		Serpentine receptor, class W	-2.33	0.0002	n.s.	n.s.	-3.64	>0.0001
	CRE21838			1.91	0.0022	2.25	0.0001	3.63	0.0002
	CRE00936	*npax-2*	N-terminal PAX (PAI domain only) protein	2.13	0.0004	3.63	>0.0001	2.68	0.0018
	CRE09422			3.63	>0.0001	2.85	0.0005	3.19	0.0009
	CRE25745			3.30	>0.0001	3.60	>0.0001	3.11	>0.0001
	CRE02652			3.57	>0.0001	1.38	0.0403	2.13	0.0004
	CRE17298			-3.56	0.0001	--	--	--	--
	CRE12966		Serpentine receptor, class W	-3.56	>0.0001	--	--	-2.77	0.0008
	CRE27097			n.s.	n.s.	3.54	0.0066	n.s.	n.s.
	CRE10586		Dehydrogenase	-1.22	>0.0001	-1.05	>0.0001	-3.52	>0.0001
	CRE04420			3.51	>0.0001	--	--	--	--
	CRE18856			-3.50	>0.0001	-2.28	>0.0001	-2.17	>0.0001
	CRE23575		MAM (Meprin, A5-protein, PTPmu) domain protein	3.36	>0.0001	3.48	>0.0001	3.09	0.0013
	CRE12497			--	--	--	--	-3.48	0.0015
	CRE03438		C-type lectin	2.47	0.0002	3.47	>0.0001	--	--
	CRE05780			-3.06	>0.0001	-3.47	0.0004	--	--
	CRE02894			3.46	>0.0001	1.36	0.0231	--	--
	CRE21368			3.45	>0.0001	2.79	0.0041	--	--
	CRE16306		PAN domain-containing protein	--	--	3.44	0.0014	--	--
	CRE23687		Threonine dehydratase catabolic-like protein	-3.43	>0.0001	-3.20	>0.0001	-1.45	0.0105
	CRE00199	*ptr-4*	Patched-related family	--	--	3.43	0.0181	--	--
	CRE08830			3.42	>0.0001	--	--	--	--
	CRE28521			2.80	>0.0001	--	--	3.42	0.0154
	CRE08701		Serpentine receptor, class Z	-3.42	>0.0001	-3.06	>0.0001	--	--

HSP	Gene ID	Gene Name	Gene Description	Ancestor Log$_2$ FC	Ancestor FDR	Control Log$_2$ FC	Control FDR	Heat Log$_2$ FC	Heat FDR
	CRE01255			3.41	0.0004	--	--	--	--
	CRE01261			3.41	0.0001	--	--	--	--
◆	CRE04918	*daf-21*	HSPC (HSP90)	3.40	>0.0001	n.s.	n.s.	2.52	0.0008
	CRE30954			-3.40	>0.0001	-3.15	>0.0001	-2.37	>0.0001
	CRE08915			-2.95	0.0336	-3.40	>0.0001	--	--
	CRE01263			3.39	0.0009	--	--	--	--
	CRE06458			3.38	>0.0001	2.74	>0.0001	2.47	>0.0001
	CRE26886		Myosin light chain kinase	3.38	>0.0001	3.05	0.0103	n.s.	n.s.
	CRE08273			3.37	>0.0001	--	--	n.s.	n.s.
	CRE17339			-1.49	0.0449	-2.97	0.0001	-3.37	>0.0001
◆	CRE00198	*hsp-3*	HSP70 protein	3.36	>0.0001	1.92	0.0384	2.44	>0.0001
	CRE00152	*clec-266*	C-type lectin	3.36	>0.0001	n.s.	n.s.	3.11	0.0478
	CRE09072			-3.35	>0.0001	-3.30	>0.0001	-1.93	0.0396
◆	CRE26138		HSP70 protein	3.34	>0.0001	n.s.	n.s.	2.15	0.0302
	CRE11953			1.74	>0.0001	1.75	0.0023	3.32	0.0003
	CRE10900	*fmo-2*	Flavin monooxygenase	-3.31	>0.0001	-2.79	>0.0001	-1.41	>0.0001
	CRE10649		Zinc finger protein	2.33	>0.0001	3.29	0.0197	2.04	0.0441
	CRE09656		AMP deaminase	1.38	0.0156	--	--	3.27	0.0006
	CRE13074	*str-96*	7-transmembrane receptor	--	--	--	--	-3.26	0.0003
	CRE06193		Ribonucleotide reductase	-3.26	0.0304	-3.19	>0.0001	-2.10	0.0016
	CRE24981			1.09	0.0011	3.25	0.0058	n.s.	n.s.
	CRE09429			3.24	>0.0001	2.39	>0.0001	2.37	0.0002
	CRE09190	*cyp-34A5*	Cytochrome p450 family protein	--	--	-3.24	0.0022	--	--
	CRE06003	*fat-5*	Fatty acid desaturase	2.14	>0.0001	3.23	0.0012	1.23	>0.0001
	CRE05507		Aspartyl protease	n.s.	n.s.	n.s.	n.s.	-3.22	>0.0001
	CRE30887			3.22	>0.0001	1.30	0.0132	n.s.	n.s.
	CRE08991			3.21	>0.0001	n.s.	n.s.	2.34	>0.0001
	CRE01717			2.19	>0.0001	2.46	0.0057	3.21	0.0009
	CRE29186			-3.20	>0.0001	-2.24	>0.0001	-1.85	0.0134

HSP	Gene ID	Gene Name	Gene Description	Ancestor Log2 FC	Ancestor FDR	Control Log2 FC	Control FDR	Heat Log2 FC	Heat FDR
	CRE15658			-3.20	>0.0001	-1.20	0.0037	--	--
	CRE01661			-2.99	>0.0001	-2.59	>0.0001	-3.20	0.0005
	CRE12737			1.25	0.0036	3.20	0.0001	n.s.	n.s.
	CRE17300	*cyp-23A1*	Cytochrome p450 family protein	2.97	>0.0001	2.59	0.0451	3.20	0.0013
	CRE13167			--	--	--	--	-3.19	>0.0001
	CRE01678			--	--	-3.19	>0.0001	--	--
	CRE19390		Ani s 1 allergen	-2.53	>0.0001	-3.18	>0.0001	-1.88	0.0028
	CRE05593			-3.01	>0.0001	-2.89	>0.0001	-3.18	>0.0001
	CRE24499			3.16	>0.0001	2.17	0.0014	2.21	0.0035
	CRE09281			-3.16	>0.0001	-2.57	>0.0001	-2.49	>0.0001
	CRE16493			-3.04	>0.0001	-2.40	>0.0001	-3.15	>0.0001
	CRE04919	*gasr-8*	Growth-arrest-specific-protein 8	3.15	>0.0001	n.s.	n.s.	n.s.	n.s.
	CRE12600			3.14	>0.0001	1.99	0.0020	n.s.	n.s.
	CRE28556			3.13	>0.0001	n.s.	n.s.	n.s.	n.s.
	CRE01305			3.13	>0.0001	--	--	--	--
	CRE10806		Cysteine-rich intestinal protein-related	-1.83	>0.0001	-1.60	0.0178	-3.12	>0.0001
	CRE17641			3.11	>0.0001	--	--	--	--
	CRE12674			-1.67	0.0356	n.s.	n.s.	-3.11	>0.0001
	CRE16136			-1.04	0.0486	-3.11	>0.0001	n.s.	n.s.
	CRE06373		Serpentine receptor, class I	3.10	>0.0001	--	--	--	--
	CRE03436		C-type lectin	2.54	>0.0001	3.09	>0.0001	1.80	0.0083
	CRE19316			--	--	3.09	0.0015	--	--
	CRE20157		Ribonucleotide reductase	--	--	-3.08	>0.0001	-1.73	0.0289
	CRE03421	*sru-7*	Serpentine receptor, class U	-3.08	>0.0001	n.s.	n.s.	--	--
	CRE01311			3.07	>0.0001	--	--	--	--
◆	CRE26406		HSP70 protein	3.07	>0.0001	2.33	0.0044	2.05	0.0075
	CRE15096		Cytochrome p450 family protein	-1.91	0.0007	-3.06	>0.0001	n.s.	n.s.
	CRE08150			3.06	>0.0001	n.s.	n.s.	n.s.	n.s.
	CRE10669			-3.05	>0.0001	-1.96	>0.0001	-3.05	>0.0001

HSP	Gene ID	Gene Name	Gene Description	Ancestor Log_2 FC	Ancestor FDR	Control Log_2 FC	Control FDR	Heat Log_2 FC	Heat FDR
	CRE02873			3.04	>0.0001	1.31	0.0283	2.90	0.0037
	CRE31451			3.03	>0.0001	--	--	--	--

*Model did not converge

Table S3 List of genes differentially expressed over evolutionary time (20°C environment). Listed are genes that were differentially expressed between the ancestor and either evolved population. Dashes indicate that the gene was not detected above our detection threshold in either of the lines under comparison; n.s. indicates that transcript levels were not significantly different between lines.

	GeneID	Gene Name	Gene Description	FC: Heat/ Ancestor	FDR (Heat)	FC: Ctrl/ Ancestor	FDR (Control)
**DE in Heat-Selected Line**	CRE23514			8.49	0.0013	--	--
	CRE24278		C-type lectin	7.78	0.0002	--	--
	CRE13167		CUB-like domain	4.32	0.0316	--	--
	CRE16108		CUB-like domain	4.03	0.0156	--	--
	CRE10142		CUB-like domain	3.19	0.0395	--	--
	CRE20697		UDP-glucuronosyl transferase	3.12	0.0316	n.s.	n.s.
	CRE18453		Molybdenum cofactor sulfurase	2.84	0.0000	n.s.	n.s.
	CRE30538		Helitron helicase-like domain	-2.79	0.0037	n.s.	n.s.
	CRE15658			-2.62	0.0012	n.s.	n.s.
	CRE10586		Short-chain dehydrogenase	2.59	0.0366	n.s.	n.s.
	CRE02477		Short-chain dehydrogenase	2.55	0.0137	n.s.	n.s.
	CRE09885			2.49	0.0034	n.s.	n.s.
	CRE24807			-2.42	0.0000	n.s.	n.s.
	CRE12164	*glb-1*	Globin-related protein	2.06	0.0000	n.s.	n.s.
	CRE28721	*lact-6*	Beta-lactamase related protein	1.94	0.0357	n.s.	n.s.
	CRE09484		C-type lectin	1.83	0.0013	n.s.	n.s.
	CRE09800		Zinc finger protein	1.79	0.0041	n.s.	n.s.
	CRE25687			-1.79	0.0026	n.s.	n.s.
	CRE11848		Glutathione S-transferase	1.73	0.0050	n.s.	n.s.
	CRE10310		SCP-like extracellular protein	-1.67	0.0318	n.s.	n.s.
	CRE18035		aminoglycoside phosphotransferase	-1.66	0.0019	n.s.	n.s.
	CRE30855		NADH oxidase	1.63	0.0006	n.s.	n.s.
	CRE10033			-1.61	0.0437	n.s.	n.s.
	CRE22864	*lipl-2*	Lipase-like protein	-1.58	0.0051	n.s.	n.s.
	CRE12163		Cytochrome b5	1.58	0.0169	n.s.	n.s.
	CRE00804	*amt-1*	Ammonium transporter homolog	-1.50	0.0312	n.s.	n.s.

	GeneID	Gene Name	Gene Description	FC: Heat/ Ancestor	FDR (Heat)	FC: Ctrl/ Ancestor	FDR (Control)
	CRE09420			1.37	0.0342	n.s.	n.s.
	CRE18856			-1.30	0.0211	n.s.	n.s.
	CRE13371	*nit-1*	Nitrilase	1.20	0.0301	n.s.	n.s.
**DE in Control Line**	CRE23798		Integrase	n.s.	n.s.	-6.11	0.0000
	CRE12053		DDE endonuclease	--	--	3.29	0.0001
	CRE16136		Methyltransferase	n.s.	n.s.	3.08	0.0479
	CRE06358			--	--	2.90	0.0014
	CRE07402		Integrase	n.s.	n.s.	-2.73	0.0000
	CRE24828			n.s.	n.s.	2.33	0.0104
	CRE19091			--	--	2.27	0.0003
	CRE00568			n.s.	n.s.	-2.26	0.0034
	CRE30234			n.s.	n.s.	2.09	0.0012
	CRE12487			n.s.	n.s.	-2.04	0.0072
	CRE08705		Glutathione S-transferase	n.s.	n.s.	2.04	0.0012
	CRE10692		Cytochrome p450 family protein	n.s.	n.s.	1.97	0.0000
	CRE18381			n.s.	n.s.	-1.94	0.0179
	CRE09193	*cyp-34A10*	Cytochrome p450 family protein	n.s.	n.s.	-1.93	0.0104
	CRE19314		Protein kinase	n.s.	n.s.	-1.89	0.0151
	CRE28667			--	--	1.88	0.0059
	CRE23839			n.s.	n.s.	1.83	0.0012
	CRE25599		Lipase-like protein	--	--	1.78	0.0381
	CRE15096		Cytochrome p450 family protein	n.s.	n.s.	1.72	0.0214
	CRE11440		Integrase	n.s.	n.s.	1.57	0.0003
	CRE25834		Integrase	n.s.	n.s.	1.50	0.0434
	CRE06192			n.s.	n.s.	1.50	0.0034
	CRE28296		Calponin	n.s.	n.s.	-1.46	0.0071
	CRE27735		Prion-like (Q/N-rich) domain protein	n.s.	n.s.	1.38	0.0068
	CRE17915		Acyl-CoA thioesterase	n.s.	n.s.	-1.36	0.0028
	CRE20531			n.s.	n.s.	1.32	0.0242

	GeneID	Gene Name	Gene Description	FC: Heat/ Ancestor	FDR (Heat)	FC: Ctrl/ Ancestor	FDR (Control)
	CRE29200			n.s.	n.s.	-1.32	0.0063
	CRE09559		5'-nucleotidase	n.s.	n.s.	1.29	0.0095
	CRE29277		SCP-like extracellular protein	n.s.	n.s.	-1.28	0.0154
	CRE10669			n.s.	n.s.	-1.26	0.0120
	CRE14147			n.s.	n.s.	1.26	0.0298
	CRE06222		Integrase	n.s.	n.s.	1.24	0.0124
	CRE09560			n.s.	n.s.	1.16	0.0179
	CRE26779		Integrase	n.s.	n.s.	-1.13	0.0428
	CRE24146			n.s.	n.s.	1.13	0.0104
	CRE29479			n.s.	n.s.	1.10	0.0154
	CRE14067			n.s.	n.s.	-1.10	0.0283
	CRE08770		Glutathione S-transferase	n.s.	n.s.	1.01	0.0136
	CRE14333		5-oxoprolinase	n.s.	n.s.	0.99	0.0198
**DE in Both Selected Lines**	CRE14636	*clec-140*	C-type lectin	6.10	0.0006	1.95	0.0000
	CRE14503		C-type lectin	5.72	0.0008	1.85	0.0000
	CRE01641	*chil-8*	Chitinase	-3.41	0.0421	-5.27	0.0001
	CRE21731			-2.84	0.0037	1.68	0.0000
	CRE05881			-1.75	0.0328	-2.67	0.0000
	CRE02474		Dehydrogenase	2.58	0.0143	1.72	0.0000
	CRE13743			2.52	0.0000	2.33	0.0000
	CRE21610		C-type lectin	-2.42	0.0136	-1.55	0.0150
	CRE09194	*nlp-34*	Neuropeptide-like protein	-2.38	0.0003	-1.74	0.0059
	CRE13741		Integrase	2.36	0.0000	2.09	0.0000
	CRE09886			2.27	0.0006	1.73	0.0179
	CRE13742			2.26	0.0026	1.95	0.0006
	CRE13476	*thn-5*	Thaumatin-like protein	-2.19	0.0000	-2.02	0.0000
	CRE29705		Integrase	2.10	0.0000	1.95	0.0000
	CRE07706			2.07	0.0000	1.40	0.0014
	CRE25992		NADH oxidase	2.03	0.0019	1.13	0.0059

GeneID	Gene Name	Gene Description	FC: Heat/ Ancestor	FDR (Heat)	FC: Ctrl/ Ancestor	FDR (Control)
CRE14226		UDP-glucuronosyl transferase	1.86	0.0001	1.44	0.0005
CRE13701			1.80	0.0006	1.40	0.0136
CRE29704			1.77	0.0036	1.66	0.0005
CRE29212	*gst-1*	Glutathione S-transferase	1.75	0.0001	1.33	0.0012
CRE29481			1.56	0.0026	1.42	0.0001
CRE07709			1.52	0.0019	1.18	0.0028

Files S1-S2

Available for download as CSV files at http://www.g3journal.org/lookup/suppl/doi:10.1534/g3.114.010553/-/DC1

File S1 Phenotypic responses for the experimental evolution lines

File S2 Data for the full heat shock reaction norm for each line
