# Supplementary material for: Rapid Evolution of Phenotypic Plasticity and Shifting Thresholds of Genetic Assimilation in the Nematode Caenorhabditis remanei
Source: G3 (Bethesda). 2014 Apr 11;4(6):1103–12. doi: 10.1534/g3.114.010553 (PMC4065253; doi:10.1534/g3.114.010553)
Supplement: Supporting Information [file supp_g3.114.010553_FigureS1.pdf]

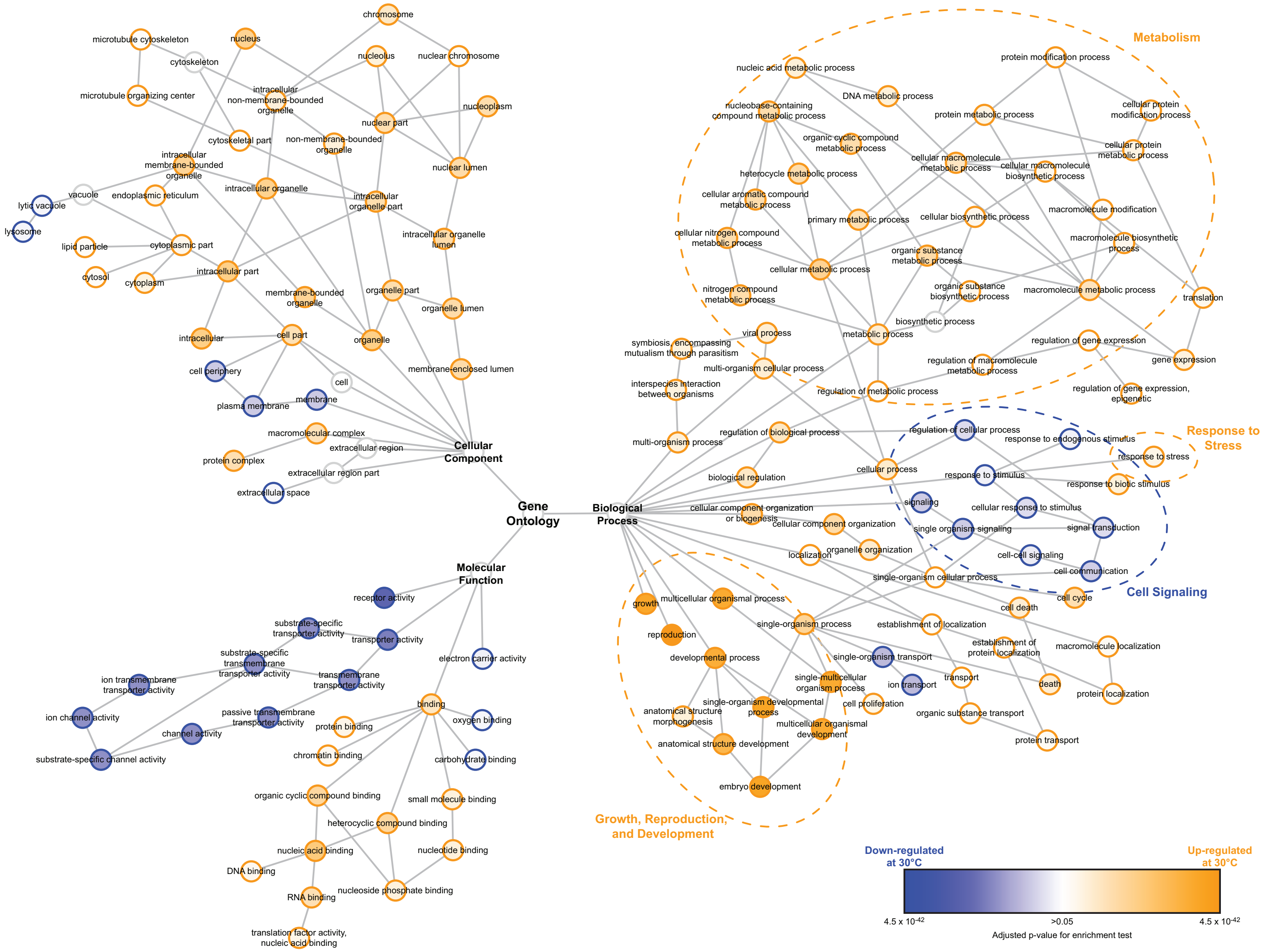

Figure S1 Gene ontology enrichment network for genes differentially expressed by environment. Shown are GOSlim terms with significant enrichment (FDR < 5%) for the up-regulated genes (orange) or down-regulated genes (blue) in the ancestral population. The intensity of shading is proportional to the significance value from the Fisher's Exact Test for the ontology term.
