# Supplementary material for: Rapid Evolution of Phenotypic Plasticity and Shifting Thresholds of Genetic Assimilation in the Nematode Caenorhabditis remanei
Source: G3 (Bethesda). 2014 Apr 11;4(6):1103–12. doi: 10.1534/g3.114.010553 (PMC4065253; doi:10.1534/g3.114.010553)
Supplement: Supporting Information [file supp_g3.114.010553_TableS1.pdf]

Table S1 Summary of differential expression results by line.

Line	Genes expressed above threshold	Differential expression (FDR 5%)
Ancestor	15,347	6431
Control	15,141	4286
Heat	14,784	2769
Combined (all lines)	15,963	8377
