# Supplementary material for: Rapid Evolution of Phenotypic Plasticity and Shifting Thresholds of Genetic Assimilation in the Nematode Caenorhabditis remanei
Source: G3 (Bethesda). 2014 Apr 11;4(6):1103–12. doi: 10.1534/g3.114.010553 (PMC4065253; doi:10.1534/g3.114.010553)
Supplement: Supporting Information [file supp_g3.114.010553_TableS2.pdf]

Table S2 List of the 200 most differentially expressed genes across environments from the RNA-seq analysis. Dashes indicate that the gene was not detected above our detection threshold at either temperature for the line under comparison; n.s. indicates that transcript levels were not significantly different between environments.

HSP	Gene ID	Gene Name	Gene Description	Ancestor Log_2 FC	Ancestor FDR	Control Log_2 FC	Control FDR	Heat Log_2 FC	Heat FDR
	CRE09388		C-type lectin	35.31	>0.0001	--	--	4.61	>0.0001
	CRE24573		Protein kinase	-5.31	>0.0001	-34.57	>0.0001	-33.46	>0.0001
	CRE08067			32.35	>0.0001	--	--	--	--
	CRE23800		Hydrolase	8.99	>0.0001	7.00	>0.0001	n.s.	n.s.
◆	CRE24849		HSP70 protein	8.66	>0.0001	7.31	0.0011	5.64	0.0017
	CRE19381			8.22	>0.0001	8.57	0.0024	5.33	0.0344
	CRE18157			6.14	>0.0001	8.52	0.0059	--	--
	CRE18318			8.50	>0.0001	--	--	6.22	0.0198
◆	CRE04868		HSP70 protein	8.05	>0.0001	6.83	0.0116	5.78	0.0006
	CRE26773		Integrase	8.03	>0.0001	6.09	0.0222	6.09	>0.0001
◆	CRE01097		HSP70 protein	7.90	>0.0001	6.50	0.0192	5.73	0.0035
◆	CRE18319		Small hsp (HSPB)	7.90	>0.0001	6.93	0.0035	5.01	0.0002
◆	CRE18317		Small hsp (HSPB)	7.86	>0.0001	5.61	>0.0001	4.97	0.0013
◆	CRE04869		HSP70 protein	7.81	>0.0001	6.79	0.0166	5.87	0.0043
◆	CRE01029		HSP70 protein	7.72	>0.0001	6.86	0.0052	5.79	0.0008
	CRE26772		Reverse transcriptase	7.57	>0.0001	n.s.	n.s.	5.99	>0.0001
◆	CRE19380		Small hsp (HSPB)	6.99	>0.0001	7.38	0.0005	5.03	>0.0001
◆	CRE18323		Small hsp (HSPB)	7.36	>0.0001	6.48	0.0048	5.56	>0.0001
◆	CRE04666		HSP70 protein	7.33	0.0017*	--	--	4.59	0.0188
◆	CRE19334		Small hsp (HSPB)	7.30	>0.0001	6.47	0.0081	5.11	>0.0001
◆	CRE26901		HSP70 protein	6.49	>0.0001	7.28	0.0043	5.54	>0.0001
◆	CRE18316		Small hsp (HSPB)	6.65	>0.0001	7.15	>0.0001	5.38	>0.0001
◆	CRE18322		Small hsp (HSPB)	7.01	>0.0001	6.96	0.0003	5.10	>0.0001
◆	CRE19384		Small hsp (HSPB)	7.01	>0.0001	6.64	0.0017	4.83	>0.0001
	CRE23104			6.95	>0.0001	--	--	--	--
◆	CRE19383		Small hsp (HSPB)	6.93	>0.0001	6.64	0.0006	5.44	>0.0001
◆	CRE25393		HSP70 protein	6.93	>0.0001	6.43	0.0035	5.50	0.0005

HSP	Gene ID	Gene Name	Gene Description	Ancestor Log_2 FC	Ancestor FDR	Control Log_2 FC	Control FDR	Heat Log_2 FC	Heat FDR
◆	CRE19333		Small hsp (HSPB)	6.61	>0.0001	6.91	0.0009	5.31	>0.0001
◆	CRE18315		Small hsp (HSPB)	6.63	>0.0001	6.75	0.0007	4.91	>0.0001
◆	CRE19335		Small hsp (HSPB)	6.74	>0.0001	6.68	>0.0001	4.75	0.0001
	CRE01030			6.62	>0.0001	--	--	6.08	>0.0001
◆	CRE18321		Small hsp (HSPB)	6.61	>0.0001	6.46	0.0004	4.93	>0.0001
◆	CRE27162		Small hsp (HSPB)	6.50	>0.0001	6.37	0.0077	5.23	0.0016
	CRE05591			n.s.	n.s.	-6.47	>0.0001	--	--
◆	CRE20780	*hsp-70*	HSP70 protein	6.42	>0.0001	6.26	0.0003	5.27	>0.0001
	CRE24278		C-type lectin	--	--	--	--	-6.39	>0.0001
◆	CRE27471		Small hsp (HSPB)	6.27	>0.0001	6.27	0.0027	5.16	0.0001
	CRE21296		Reverse transcriptase	6.13	>0.0001	n.s.	n.s.	6.18	>0.0001
	CRE16108		CUB-like domain	--	--	--	--	-6.07	>0.0001
	CRE05459	*end-3*	GATA zinc finger transcription factor	6.05	>0.0001	--	--	--	--
	CRE20711		UDP-glucose:glycoprotein glucosyltransferase	5.87	>0.0001	n.s.	n.s.	4.61	0.0010
◆	CRE19382		Small hsp (HSPB)	5.80	>0.0001	5.22	>0.0001	4.99	0.0018
	CRE27833		Helitron helicase-like domain	5.69	>0.0001	--	--	n.s.	n.s.
	CRE19026		Metridin-like ShK toxin domain	-5.06	>0.0001	-5.51	>0.0001	-5.62	>0.0001
	CRE06466	*sre-42*	Serpentine receptor, class E	-3.61	0.0001	-2.54	0.0028	-5.45	>0.0001
	CRE10142		CUB-like domain	--	--	--	--	-5.39	>0.0001
	CRE11034			5.38	>0.0001	3.81	>0.0001	1.33	>0.0001
	CRE28993		Serpentine receptor, class W	--	--	5.38	>0.0001	--	--
	CRE08692		Serpentine receptor, class Z	--	--	--	--	-5.37	>0.0001
	CRE22721			5.34	>0.0001	--	--	5.10	0.0022
	CRE16387			3.95	>0.0001	3.37	>0.0001	5.24	0.0055
	CRE28585			4.66	>0.0001	n.s.	n.s.	4.19	0.0016
	CRE27404	*gcy-13*	Guanylate cyclase	-1.46	0.0301	--	--	-5.14	0.0015
	CRE24995	*cdh-7*	Cadherin	--	--	4.99	0.0314	--	--
	CRE08101			4.68	>0.0001	--	--	--	--

HSP	Gene ID	Gene Name	Gene Description	Ancestor Log$_2$ FC	Ancestor FDR	Control Log$_2$ FC	Control FDR	Heat Log$_2$ FC	Heat FDR
	CRE09372			3.44	>0.0001	4.64	0.0489	2.87	>0.0001
	CRE20636	*phy-2*	Prolyl 4-hydroxylase	4.52	>0.0001	4.61	>0.0001	2.03	>0.0001
	CRE01098			1.77	>0.0001	n.s.	n.s.	4.59	>0.0001
	CRE10141			--	--	--	--	-4.57	>0.0001
	CRE03576		Hydrolase	3.77	>0.0001	4.55	>0.0001	2.01	>0.0001
	CRE12322			--	--	--	--	4.53	0.0016
	CRE14503		C-type lectin	n.s.	n.s.	-1.38	>0.0001	-4.53	>0.0001
	CRE14636	*clec-140*	C-type lectin	n.s.	n.s.	-1.23	0.0004	-4.51	>0.0001
	CRE09419			4.45	>0.0001	2.64	0.0224	4.50	>0.0001
	CRE08905			-3.46	0.0009	-4.48	>0.0001	--	--
	CRE20697		Hexosyltransferase	n.s.	n.s.	-1.26	0.0003	-4.46	>0.0001
	CRE13476		Thaumatin-like protein	-4.46	>0.0001	-3.03	>0.0001	-3.64	>0.0001
	CRE29499			4.44	>0.0001	--	--	3.55	0.0004
	CRE08033			4.43	>0.0001	--	--	--	--
	CRE23366			4.38	>0.0001	--	--	--	--
	CRE11169			4.36	>0.0001	2.78	0.0011	2.12	0.0411
	CRE24133		Collagen	n.s.	n.s.	-4.36	>0.0001	-2.32	>0.0001
	CRE02480		Nuclear hormone receptor	--	--	-1.70	0.0219	-4.35	0.0001
	CRE16040			2.38	>0.0001	4.28	>0.0001	2.55	>0.0001
	CRE03584			4.24	>0.0001	--	--	3.38	0.0061
	CRE31291			4.22	>0.0001	3.03	0.0043	3.01	0.0053
	CRE24819			4.21	>0.0001	2.84	0.0062	2.04	>0.0001
	CRE01842	*clec-60*	C-type lectin	3.71	>0.0001	4.20	>0.0001	n.s.	n.s.
	CRE07273		Lipocalin-related protein	n.s.	n.s.	4.13	0.0461	1.69	0.0140
	CRE26387			--	--	4.11	0.0054	--	--
	CRE13045		Metridin-like ShK toxin domain	-4.09	>0.0001	-4.10	>0.0001	--	--
	CRE15564		Prolyl 4-hydroxylase	3.97	>0.0001	4.08	>0.0001	2.31	>0.0001
	CRE23551			3.28	>0.0001	--	--	4.07	>0.0001
	CRE06358			--	--	-4.07	>0.0001	--	--

HSP	Gene ID	Gene Name	Gene Description	Ancestor Log_2 FC	Ancestor FDR	Control Log_2 FC	Control FDR	Heat Log_2 FC	Heat FDR
	CRE20525		Serpentine receptor, class E	--	--	--	--	4.07	0.0104
	CRE13953		Fatty acid CoA synthetase family	2.49	>0.0001	2.06	0.0001	4.06	0.0001
	CRE08193			--	--	2.15	0.0266	4.04	0.0130
	CRE21837			2.36	>0.0001	2.52	0.0025	4.01	0.0001
	CRE01268			4.01	>0.0001	1.35	0.0356	2.46	0.0345
	CRE18121	*mlt-10*		4.01	>0.0001	3.68	>0.0001	1.42	>0.0001
	CRE06617		Serpentine receptor, class I	3.98	>0.0001	--	--	--	--
	CRE01964			3.96	>0.0001	3.50	>0.0001	2.23	>0.0001
	CRE01322			3.95	>0.0001	--	--	--	--
	CRE01264			3.95	>0.0001	--	--	--	--
	CRE30275			-2.09	0.0018	-3.88	>0.0001	-2.54	>0.0001
	CRE18513			-3.88	>0.0001	-2.10	>0.0001	-3.19	>0.0001
	CRE13168			--	--	--	--	-3.86	>0.0001
	CRE01279			3.86	>0.0001	1.96	0.0010	n.s.	n.s.
	CRE01306			3.86	>0.0001	n.s.	n.s.	--	--
	CRE22946			-3.84	>0.0001	-2.11	0.0017	--	--
	CRE18158			3.21	>0.0001	n.s.	n.s.	n.s.	n.s.
	CRE08879	*srh-129*	Serpentine receptor, class H	-3.83	0.0003	--	--	-2.50	0.0265
	CRE11248	*srx-85*	Serpentine receptor, class X	3.81	>0.0001	--	--		
	CRE02474			-2.87	>0.0001	-2.74	>0.0001	-3.81	>0.0001
	CRE09165			-3.77	>0.0001	-1.71	0.0006	--	--
	CRE30010	*ech-9*	Enoyl-CoA hydratase	1.91	0.0003	3.76	>0.0001	n.s.	n.s.
	CRE05592			n.s.	n.s.	-3.49	>0.0001	-3.74	>0.0001
	CRE30392			3.73	>0.0001	n.s.	n.s.	n.s.	n.s.
	CRE21032		Flavin monooxygenase	-3.73	>0.0001	-2.79	>0.0001	-1.64	>0.0001
	CRE18358			3.03	>0.0001	--	--	3.73	0.0001
	CRE09421			3.69	>0.0001	3.11	>0.0001	3.11	>0.0001
	CRE03133			-3.69	>0.0001	-1.36	>0.0001	-2.38	0.0226
	CRE17248	*aagr-4*	Acid alpha glucosidase related	2.40	>0.0001	3.68	>0.0001	2.18	>0.0001

HSP	Gene ID	Gene Name	Gene Description	Ancestor Log_2 FC	Ancestor FDR	Control Log_2 FC	Control FDR	Heat Log_2 FC	Heat FDR
	CRE01319			3.68	>0.0001	2.31	0.0023	n.s.	n.s.
	CRE09022			-3.45	>0.0001	-3.66	>0.0001	-2.64	0.0007
	CRE03585			3.65	>0.0001	n.s.	n.s.	3.60	0.0027
	CRE03432		Serpentine receptor, class W	-2.33	0.0002	n.s.	n.s.	-3.64	>0.0001
	CRE21838			1.91	0.0022	2.25	0.0001	3.63	0.0002
	CRE00936	*npax-2*	N-terminal PAX (PAI domain only) protein	2.13	0.0004	3.63	>0.0001	2.68	0.0018
	CRE09422			3.63	>0.0001	2.85	0.0005	3.19	0.0009
	CRE25745			3.30	>0.0001	3.60	>0.0001	3.11	>0.0001
	CRE02652			3.57	>0.0001	1.38	0.0403	2.13	0.0004
	CRE17298			-3.56	0.0001	--	--	--	--
	CRE12966		Serpentine receptor, class W	-3.56	>0.0001	--	--	-2.77	0.0008
	CRE27097			n.s.	n.s.	3.54	0.0066	n.s.	n.s.
	CRE10586		Dehydrogenase	-1.22	>0.0001	-1.05	>0.0001	-3.52	>0.0001
	CRE04420			3.51	>0.0001	--	--	--	--
	CRE18856			-3.50	>0.0001	-2.28	>0.0001	-2.17	>0.0001
	CRE23575		MAM (Meprin, A5-protein, PTPmu) domain protein	3.36	>0.0001	3.48	>0.0001	3.09	0.0013
	CRE12497			--	--	--	--	-3.48	0.0015
	CRE03438		C-type lectin	2.47	0.0002	3.47	>0.0001	--	--
	CRE05780			-3.06	>0.0001	-3.47	0.0004	--	--
	CRE02894			3.46	>0.0001	1.36	0.0231	--	--
	CRE21368			3.45	>0.0001	2.79	0.0041	--	--
	CRE16306		PAN domain-containing protein	--	--	3.44	0.0014	--	--
	CRE23687		Threonine dehydratase catabolic-like protein	-3.43	>0.0001	-3.20	>0.0001	-1.45	0.0105
	CRE00199	*ptr-4*	Patched-related family	--	--	3.43	0.0181	--	--
	CRE08830			3.42	>0.0001	--	--	--	--
	CRE28521			2.80	>0.0001	--	--	3.42	0.0154
	CRE08701		Serpentine receptor, class Z	-3.42	>0.0001	-3.06	>0.0001	--	--

HSP	Gene ID	Gene Name	Gene Description	Ancestor Log_2 FC	Ancestor FDR	Control Log_2 FC	Control FDR	Heat Log_2 FC	Heat FDR
	CRE01255			3.41	0.0004	--	--	--	--
	CRE01261			3.41	0.0001	--	--	--	--
◆	CRE04918	*daf-21*	HSPC (HSP90)	3.40	>0.0001	n.s.	n.s.	2.52	0.0008
	CRE30954			-3.40	>0.0001	-3.15	>0.0001	-2.37	>0.0001
	CRE08915			-2.95	0.0336	-3.40	>0.0001	--	--
	CRE01263			3.39	0.0009	--	--	--	--
	CRE06458			3.38	>0.0001	2.74	>0.0001	2.47	>0.0001
	CRE26886		Myosin light chain kinase	3.38	>0.0001	3.05	0.0103	n.s.	n.s.
	CRE08273			3.37	>0.0001	--	--	n.s.	n.s.
	CRE17339			-1.49	0.0449	-2.97	0.0001	-3.37	>0.0001
◆	CRE00198	*hsp-3*	HSP70 protein	3.36	>0.0001	1.92	0.0384	2.44	>0.0001
	CRE00152	*clec-266*	C-type lectin	3.36	>0.0001	n.s.	n.s.	3.11	0.0478
	CRE09072			-3.35	>0.0001	-3.30	>0.0001	-1.93	0.0396
◆	CRE26138		HSP70 protein	3.34	>0.0001	n.s.	n.s.	2.15	0.0302
	CRE11953			1.74	>0.0001	1.75	0.0023	3.32	0.0003
	CRE10900	*fmo-2*	Flavin monooxygenase	-3.31	>0.0001	-2.79	>0.0001	-1.41	>0.0001
	CRE10649		Zinc finger protein	2.33	>0.0001	3.29	0.0197	2.04	0.0441
	CRE09656		AMP deaminase	1.38	0.0156	--	--	3.27	0.0006
	CRE13074	*str-96*	7-transmembrane receptor	--	--	--	--	-3.26	0.0003
	CRE06193		Ribonucleotide reductase	-3.26	0.0304	-3.19	>0.0001	-2.10	0.0016
	CRE24981			1.09	0.0011	3.25	0.0058	n.s.	n.s.
	CRE09429			3.24	>0.0001	2.39	>0.0001	2.37	0.0002
	CRE09190	*cyp-34A5*	Cytochrome p450 family protein	--	--	-3.24	0.0022	--	--
	CRE06003	*fat-5*	Fatty acid desaturase	2.14	>0.0001	3.23	0.0012	1.23	>0.0001
	CRE05507		Aspartyl protease	n.s.	n.s.	n.s.	n.s.	-3.22	>0.0001
	CRE30887			3.22	>0.0001	1.30	0.0132	n.s.	n.s.
	CRE08991			3.21	>0.0001	n.s.	n.s.	2.34	>0.0001
	CRE01717			2.19	>0.0001	2.46	0.0057	3.21	0.0009
	CRE29186			-3.20	>0.0001	-2.24	>0.0001	-1.85	0.0134

HSP	Gene ID	Gene Name	Gene Description	Ancestor Log_2 FC	Ancestor FDR	Control Log_2 FC	Control FDR	Heat Log_2 FC	Heat FDR
	CRE15658			-3.20	>0.0001	-1.20	0.0037	--	--
	CRE01661			-2.99	>0.0001	-2.59	>0.0001	-3.20	0.0005
	CRE12737			1.25	0.0036	3.20	0.0001	n.s.	n.s.
	CRE17300	*cyp-23A1*	Cytochrome p450 family protein	2.97	>0.0001	2.59	0.0451	3.20	0.0013
	CRE13167			--	--	--	--	-3.19	>0.0001
	CRE01678			--	--	-3.19	>0.0001	--	--
	CRE19390		Ani s 1 allergen	-2.53	>0.0001	-3.18	>0.0001	-1.88	0.0028
	CRE05593			-3.01	>0.0001	-2.89	>0.0001	-3.18	>0.0001
	CRE24499			3.16	>0.0001	2.17	0.0014	2.21	0.0035
	CRE09281			-3.16	>0.0001	-2.57	>0.0001	-2.49	>0.0001
	CRE16493			-3.04	>0.0001	-2.40	>0.0001	-3.15	>0.0001
	CRE04919	*gasr-8*	Growth-arrest-specific-protein 8	3.15	>0.0001	n.s.	n.s.	n.s.	n.s.
	CRE12600			3.14	>0.0001	1.99	0.0020	n.s.	n.s.
	CRE28556			3.13	>0.0001	n.s.	n.s.	n.s.	n.s.
	CRE01305			3.13	>0.0001	--	--	--	--
	CRE10806		Cysteine-rich intestinal protein-related	-1.83	>0.0001	-1.60	0.0178	-3.12	>0.0001
	CRE17641			3.11	>0.0001	--	--	--	--
	CRE12674			-1.67	0.0356	n.s.	n.s.	-3.11	>0.0001
	CRE16136			-1.04	0.0486	-3.11	>0.0001	n.s.	n.s.
	CRE06373		Serpentine receptor, class I	3.10	>0.0001	--	--	--	--
	CRE03436		C-type lectin	2.54	>0.0001	3.09	>0.0001	1.80	0.0083
	CRE19316			--	--	3.09	0.0015	--	--
	CRE20157		Ribonucleotide reductase	--	--	-3.08	>0.0001	-1.73	0.0289
	CRE03421	*sru-7*	Serpentine receptor, class U	-3.08	>0.0001	n.s.	n.s.	--	--
	CRE01311			3.07	>0.0001	--	--	--	--
◆	CRE26406		HSP70 protein	3.07	>0.0001	2.33	0.0044	2.05	0.0075
	CRE15096		Cytochrome p450 family protein	-1.91	0.0007	-3.06	>0.0001	n.s.	n.s.
	CRE08150			3.06	>0.0001	n.s.	n.s.	n.s.	n.s.
	CRE10669			-3.05	>0.0001	-1.96	>0.0001	-3.05	>0.0001

HSP	Gene ID	Gene Name	Gene Description	Ancestor Log_2 FC	Ancestor FDR	Control Log_2 FC	Control FDR	Heat Log_2 FC	Heat FDR
	CRE02873			3.04	>0.0001	1.31	0.0283	2.90	0.0037
	CRE31451			3.03	>0.0001	--	--	--	--

*Model did not converge
