# Supplementary material for: Rapid Evolution of Phenotypic Plasticity and Shifting Thresholds of Genetic Assimilation in the Nematode Caenorhabditis remanei
Source: G3 (Bethesda). 2014 Apr 11;4(6):1103–12. doi: 10.1534/g3.114.010553 (PMC4065253; doi:10.1534/g3.114.010553)
Supplement: Supporting Information [file supp_g3.114.010553_TableS3.pdf]

Table S3 List of genes differentially expressed over evolutionary time (20°C environment). Listed are genes that were differentially expressed between the ancestor and either evolved population. Dashes indicate that the gene was not detected above our detection threshold in either of the lines under comparison; n.s. indicates that transcript levels were not significantly different between lines.

	GeneID	Gene Name	Gene Description	FC: Heat/ Ancestor	FDR (Heat)	FC: Ctrl/ Ancestor	FDR (Control)
**DE in Heat-Selected Line**	CRE23514			8.49	0.0013	--	--
	CRE24278		C-type lectin	7.78	0.0002	--	--
	CRE13167		CUB-like domain	4.32	0.0316	--	--
	CRE16108		CUB-like domain	4.03	0.0156	--	--
	CRE10142		CUB-like domain	3.19	0.0395	--	--
	CRE20697		UDP-glucuronosyl transferase	3.12	0.0316	n.s.	n.s.
	CRE18453		Molybdenum cofactor sulfurase	2.84	0.0000	n.s.	n.s.
	CRE30538		Helitron helicase-like domain	-2.79	0.0037	n.s.	n.s.
	CRE15658			-2.62	0.0012	n.s.	n.s.
	CRE10586		Short-chain dehydrogenase	2.59	0.0366	n.s.	n.s.
	CRE02477		Short-chain dehydrogenase	2.55	0.0137	n.s.	n.s.
	CRE09885			2.49	0.0034	n.s.	n.s.
	CRE24807			-2.42	0.0000	n.s.	n.s.
	CRE12164	*glb-1*	Globin-related protein	2.06	0.0000	n.s.	n.s.
	CRE28721	*lact-6*	Beta-lactamase related protein	1.94	0.0357	n.s.	n.s.
	CRE09484		C-type lectin	1.83	0.0013	n.s.	n.s.
	CRE09800		Zinc finger protein	1.79	0.0041	n.s.	n.s.
	CRE25687			-1.79	0.0026	n.s.	n.s.
	CRE11848		Glutathione S-transferase	1.73	0.0050	n.s.	n.s.
	CRE10310		SCP-like extracellular protein	-1.67	0.0318	n.s.	n.s.
	CRE18035		aminoglycoside phosphotransferase	-1.66	0.0019	n.s.	n.s.
	CRE30855		NADH oxidase	1.63	0.0006	n.s.	n.s.
	CRE10033			-1.61	0.0437	n.s.	n.s.
	CRE22864	*lipl-2*	Lipase-like protein	-1.58	0.0051	n.s.	n.s.
	CRE12163		Cytochrome b5	1.58	0.0169	n.s.	n.s.
	CRE00804	*amt-1*	Ammonium transporter homolog	-1.50	0.0312	n.s.	n.s.

	GeneID	Gene Name	Gene Description	FC: Heat/ Ancestor	FDR (Heat)	FC: Ctrl/ Ancestor	FDR (Control)
	CRE09420			1.37	0.0342	n.s.	n.s.
	CRE18856			-1.30	0.0211	n.s.	n.s.
	CRE13371	*nit-1*	Nitrilase	1.20	0.0301	n.s.	n.s.
**DE in Control Line**	CRE23798		Integrase	n.s.	n.s.	-6.11	0.0000
	CRE12053		DDE endonuclease	--	--	3.29	0.0001
	CRE16136		Methyltransferase	n.s.	n.s.	3.08	0.0479
	CRE06358			--	--	2.90	0.0014
	CRE07402		Integrase	n.s.	n.s.	-2.73	0.0000
	CRE24828			n.s.	n.s.	2.33	0.0104
	CRE19091			--	--	2.27	0.0003
	CRE00568			n.s.	n.s.	-2.26	0.0034
	CRE30234			n.s.	n.s.	2.09	0.0012
	CRE12487			n.s.	n.s.	-2.04	0.0072
	CRE08705		Glutathione S-transferase	n.s.	n.s.	2.04	0.0012
	CRE10692		Cytochrome p450 family protein	n.s.	n.s.	1.97	0.0000
	CRE18381			n.s.	n.s.	-1.94	0.0179
	CRE09193	*cyp-34A10*	Cytochrome p450 family protein	n.s.	n.s.	-1.93	0.0104
	CRE19314		Protein kinase	n.s.	n.s.	-1.89	0.0151
	CRE28667			--	--	1.88	0.0059
	CRE23839			n.s.	n.s.	1.83	0.0012
	CRE25599		Lipase-like protein	--	--	1.78	0.0381
	CRE15096		Cytochrome p450 family protein	n.s.	n.s.	1.72	0.0214
	CRE11440		Integrase	n.s.	n.s.	1.57	0.0003
	CRE25834		Integrase	n.s.	n.s.	1.50	0.0434
	CRE06192			n.s.	n.s.	1.50	0.0034
	CRE28296		Calponin	n.s.	n.s.	-1.46	0.0071
	CRE27735		Prion-like (Q/N-rich) domain protein	n.s.	n.s.	1.38	0.0068
	CRE17915		Acyl-CoA thioesterase	n.s.	n.s.	-1.36	0.0028
	CRE20531			n.s.	n.s.	1.32	0.0242

	GeneID	Gene Name	Gene Description	FC: Heat/ Ancestor	FDR (Heat)	FC: Ctrl/ Ancestor	FDR (Control)
	CRE29200			n.s.	n.s.	-1.32	0.0063
	CRE09559		5'-nucleotidase	n.s.	n.s.	1.29	0.0095
	CRE29277		SCP-like extracellular protein	n.s.	n.s.	-1.28	0.0154
	CRE10669			n.s.	n.s.	-1.26	0.0120
	CRE14147			n.s.	n.s.	1.26	0.0298
	CRE06222		Integrase	n.s.	n.s.	1.24	0.0124
	CRE09560			n.s.	n.s.	1.16	0.0179
	CRE26779		Integrase	n.s.	n.s.	-1.13	0.0428
	CRE24146			n.s.	n.s.	1.13	0.0104
	CRE29479			n.s.	n.s.	1.10	0.0154
	CRE14067			n.s.	n.s.	-1.10	0.0283
	CRE08770		Glutathione S-transferase	n.s.	n.s.	1.01	0.0136
	CRE14333		5-oxoprolinase	n.s.	n.s.	0.99	0.0198
**DE in Both Selected Lines**	CRE14636	*clec-140*	C-type lectin	6.10	0.0006	1.95	0.0000
	CRE14503		C-type lectin	5.72	0.0008	1.85	0.0000
	CRE01641	*chil-8*	Chitinase	-3.41	0.0421	-5.27	0.0001
	CRE21731			-2.84	0.0037	1.68	0.0000
	CRE05881			-1.75	0.0328	-2.67	0.0000
	CRE02474		Dehydrogenase	2.58	0.0143	1.72	0.0000
	CRE13743			2.52	0.0000	2.33	0.0000
	CRE21610		C-type lectin	-2.42	0.0136	-1.55	0.0150
	CRE09194	*nlp-34*	Neuropeptide-like protein	-2.38	0.0003	-1.74	0.0059
	CRE13741		Integrase	2.36	0.0000	2.09	0.0000
	CRE09886			2.27	0.0006	1.73	0.0179
	CRE13742			2.26	0.0026	1.95	0.0006
	CRE13476	*thn-5*	Thaumatin-like protein	-2.19	0.0000	-2.02	0.0000
	CRE29705		Integrase	2.10	0.0000	1.95	0.0000
	CRE07706			2.07	0.0000	1.40	0.0014
	CRE25992		NADH oxidase	2.03	0.0019	1.13	0.0059

GeneID	Gene Name	Gene Description	FC: Heat/ Ancestor	FDR (Heat)	FC: Ctrl/ Ancestor	FDR (Control)
CRE14226		UDP-glucuronosyl transferase	1.86	0.0001	1.44	0.0005
CRE13701			1.80	0.0006	1.40	0.0136
CRE29704			1.77	0.0036	1.66	0.0005
CRE29212	*gst-1*	Glutathione S-transferase	1.75	0.0001	1.33	0.0012
CRE29481			1.56	0.0026	1.42	0.0001
CRE07709			1.52	0.0019	1.18	0.0028
